# Peripheral and central auditory dysfunction, cardiometabolic multimorbidity, and cognitive performance in community-dwelling older adults: a cross-sectional study

**DOI:** 10.3389/fnins.2025.1646313

**Published:** 2026-01-16

**Authors:** Jian Ruan, Xiuhua Hu, Min Zhang, Weibin Zhang, Yan Zhang, Zhao Han, Jie Chen, Qingwei Ruan, Jingchun He, Bing Chen, Zhijun Bao

**Affiliations:** 1 Department of Otorhinolaryngology Head and Neck Surgery, Huadong Hospital Affiliated with Fudan University, Shanghai, China; 2 Shanghai Key Laboratory of Clinical Geriatrics, Shanghai Institute of Geriatrics and Gerontology, Huadong Hospital Affiliated with Shanghai Medical College, Fudan University, Shanghai, China; 3 Department of Geriatrics, Huadong Hospital Affiliated with Fudan University, Shanghai, China; 4 Laboratory of Aging, Anti-Aging & Cognitive Performance, Shanghai Institute of Geriatrics and Gerontology, Huadong Hospital Affiliated with Fudan University, Shanghai, China; 5 Department of Otorhinolaryngology Head and Neck Surgery, Xinhua Hospital, Shanghai Jiaotong University School of Medicine, Shanghai, China; 6 State Key Laboratory of Medical Neurobiology and MOE Frontiers Center for Brain Science, Department of Otorhinolaryngology, Eye & ENT Hospital, ENT Institute, Fudan University, Shanghai, China

**Keywords:** age-related hearing loss, cardiometabolic multimorbidity, central auditory processing, cognitive impairment, high-frequency hearing loss, low-frequency hearing loss

## Abstract

**Objectives:**

Both age-related peripheral or central hearing loss, and cardiometabolic multimorbidity (CMM), which are independent association with global and domain-specific cognitive impairment, are common among older adults. Cardiometabolic diseases also are independent risk factors of age-related hearing loss. The first aim of the study was to investigate the independent and joint influence of CMM and low- and high-frequency hearing loss or central auditory processing dysfunction (CAPD) on global and domain-specific cognitive impairment. The second aim was to investigate whether CMM mediate the effects of age-related hearing loss on cognitive performance.

**Methods:**

In total, 508 eligible community-dwelling dementia-free older adult participants agreed to participate and completed a cross-sectional investigation. The averages of thresholds at 0.5, 1, and 2 kHz for low frequency (LPTA) and at 4, 6, and 8 kHz for high frequency (HPTA) were calculated. CAPD was assessed using SNR (signal-to-noise ratio threshold) in a words-in-noise test. Global and domain-specific cognitive performance was measured using a comprehensive neuropsychological test battery. This study analyzed the independent associations between LPTA, HPTA, CAPD, or CMM and global and domain-specific cognitive performance after adjusting for each other and other confounders. Weighted logistic regression were used to assess the joint effects of CMM and the LPTA, HPTA, or CAPD on cognitive performance. The R package “Mediation” was used to examine whether CMM mediated the associations between LPTA, HPTA, or CAPD and cognitive performance.

**Results:**

CMM was independently associated with global cognitive performance in pre-MCI [*β* (95% CI): 0.124 (0.047, 0.202), adjusted *p* = 0.0068], MCI groups [0.131 (0.055, 0.206), adjusted *p* = 0.068] for total sample, and the sensitivity test (adjusted *p* = 0.0506, and 0.012, respectively) after adjusted for all confounders. CMM in Model 2 was also significantly associated with executive function in the sensitivity test (*β*, 0.087; 95% CI, 0.028, 0.145; adjusted *p* = 0.035). The SNR value and global cognition in Model 2 was significantly associated between the cognitively normal group and the MCI group (adjusted *p* = 0.044 in total sample, and *p* = 0.051 in sensitivity test). HPTA in Model 2 remained independently associated with attention/executive function in the sensitivity test (*β*, 0.005; 95% CI, 0.001, 0.008; adjusted *p* = 0.0395). The dose–response relationships between the LPTA, HPTA, or SNR and CMM on global cognition were most significant in the cognitively normal group than in the MCI group. The significant joint effect of CMM and HPTA on executive function also been observed. In the sensitivity test, the indirect mediation effect of HPTA on global cognitive performance in the MCI group vs. the cognitively normal group after adjustments for all confounders through CMM were significant. Approximately 16.172% of the total effect of HPTA on global cognition was explained by the mediation effect through CMM.

**Conclusion:**

CMM and CAPD were significantly associated with global cognition. CMM and HPTA were significantly associated executive function in the sensitivity test. CMM, and LPTA, HPTA, or CAPD had jointly effects on global cognition. CMM and HPTA had significant joint effect on executive function. CMM might mediate the association between the HPTA and global or executive function in individuals with LPTA ≤ 40 dB HL. These findings indicated that an integrated interventional approach for presbycusis and CMM simultaneously may delay cognitive decline in older adults.

## Introduction

Age-related hearing loss (ARHL) is the most common sensory deficit in the older population and is characterized by a loss of hearing sensitivity first observed at high frequencies and difficulty understanding speech in the presence of background noise (Gates and Mills, 2005). Deficits in the processing of auditory signals in the central nervous system and the inability to understand speech in noisy environments, but with normal hearing thresholds in some individuals, are defined as central auditory processing disorders (Bellis and Bellis, 2015; Sardone et al., 2019). An estimated 1.57 billion people globally experienced hearing loss in 2019, and 62.1% were older than 50 years. In 2050, the number of hearing-impaired people will increase 56.1% from 2019 (GBD 2019 Hearing Loss Collaborators, 2021). The prevalence in East Asia will increase 62.3% due to population aging and growth in 2036 (Huang et al., 2025). Like the prevalence of peripheral presbycusis, the prevalence of central auditory processing dysfunction (CAPD) increases with age, with approximately 95% of people over the age of 80 years demonstrating signs of central presbycusis (Stach et al., 1990; Sardone et al., 2020).

Peripheral ARHL has been identified as a modifiable risk factor, with the highest population attributable fraction for the development of dementia (Livingston et al., 2020). Over the past few decades, cumulative evidence from cross-sectional and longitudinal population-based studies has indicated that peripheral ARHL is associated with global cognitive impairment (Lin et al., 2011; Zhang et al., 2022; Wang et al., 2022), cognitive decline (Lin et al., 2013; Croll et al., 2021; Stickel et al., 2024; Samelli et al., 2025), domain-specific cognitive decline (Nicholas et al., 2021; Stickel et al., 2024), and late-life cognitive disorders (Heywood et al., 2017; Ford et al., 2018). However, a non-significant association between peripheral ARHL and global cognitive performance had also been reported in a longitudinal cohort study (Dhanda et al., 2025). Peripheral low-frequency hearing loss based on a three-frequency (0.5 kHz, 1 kHz, and 2 kHz) pure tone average (PTA) is significantly related to global cognitive status and domain-specific cognitive function, including memory, executive function, and processing speed (Harrison Bush et al., 2015; Xu et al., 2021). High-frequency hearing loss based on a two-frequency (4 and 8 kHz) PTA is significantly associated with cognitive impairment in females (Wang et al., 2022), increased risk of motoric cognitive risk syndrome and slow speed (Zhang et al., 2024), reduced performance in spatial working memory (Nicholas et al., 2021), and language and abstract abilities (Diao et al., 2021). Another study performed in rural areas of China revealed that high-frequency hearing loss based on a three-frequency PTA (3, 4, and 8 kHz) was associated with global and domain-specific cognitive impairment (Xu et al., 2021). The PTAs of three high frequencies (4, 6, and 8 kHz) were also significantly associated with spatial working memory delayed matching in a computerized neurosychological test battery (Fu et al., 2021).

Central ARHL is characterized by difficulties in auditory perception and speed communication in noisy environments due to central auditory processing dysfunction (CAPD), including temporal and frequency discrimination and binaural processing. CAPD is common in patients with Alzheimer’s disease (AD) and is a sign of mild memory impairment or subtle cognitive decline (preclinical AD) (Gates et al., 2008; Sardone et al., 2020). CAPD scores are associated with cerebrospinal fluid tau levels, entorhinal and hippocampal cortex volumes, and cognitive deficits based on a battery of neuropsychological tests and may serve as novel biomarkers for preclinical AD (Tuwaig et al., 2017). CAPD is associated with poor cognitive processing ability (Moore et al., 2014) and executive function in cognitively normal older people (Gates et al., 2010), but inconsistent results have also been reported (Mamo et al., 2019). Binaural integration dysfunction, as assessed using the Dichotic Digits Test, has been observed in preclinical AD patients with normal cognition and cerebral beta-amyloid deposition (Byun et al., 2023). Temporal resolution dysfunction has been reported in MCI patients and may indirectly reflect left temporal cortical thinning related to the transition between MCI and AD (Iliadou et al., 2017). Several longitudinal studies have shown that CAPD, as demonstrated through speech-in-noise testing or competing speech, is significantly associated with a greater risk of developing AD dementia (Gates et al., 2011).

In addition to hearing impairment, several age-related chronic diseases, including hypertension, obesity, diabetes, and depression, are also common modifiable risk factors for dementia (Livingston et al., 2020). Hypertension, diabetes, heart disease, and stroke, defined as cardiometabolic diseases (CMDs), are independent risk factors for dementia (Barbiellini Amidei et al., 2021; Wolters et al., 2018; Craig et al., 2022). The coexistence of two or more CMDs, referred to as cardiometabolic multimorbidity (CMM), is rapidly increasing among older adults (Salive, 2013). In a cross-cultural study using four geographically diverse cohorts, an increasing number of CMMs were associated with global cognitive decline in individuals without dementia in a dose-dependent manner (Jin et al., 2023). CMM is independently related to an increased risk of dementia, and genetic factors may underpin this association (Dove et al., 2023). Individuals with CMM are more than three times more likely to have dementia than those with a high genetic risk (Tai et al., 2022). CMM is associated with increased levels of tau phosphorylation and total tau (neurofibrillary tangle pathology and neuronal injury) in the cerebrospinal fluid of cognitively normal adults (Li Q. Y. et al., 2024). Apart from the association with cognitive performance, CMDs, such as hypertiosion (Lin et al., 2016), type 2 diabetes (Kim et al., 2017), and cardiovascular (Agrawal et al., 2008; Wattamwar et al., 2018) have been associated with hearing loss. The neurobiological basis of the combined effect between auditory dysfunction and CMM on cognition might involve in vascular mechanisms, neuroinflammation, and metabolic mechanisms. However, it remains largely unknown whether coexposure to CMM and ARHL may amplify and mediate the effects of ARHL on global and domain-specific cognitive performance.

In this study, we divided participants into CMD-free, single CMD, and CMM groups on the basis of CMD status. We then performed (1) to investigate the independent associations of individual CMDs, CMM, peripheral (low-frequency and high-frequency) hearing loss and CAP dysfunction with overall and domain-specific cognitive impairment in community-dwelling older participants; (2) assessments of the joint influence of peripheral or CAP dysfunction and CMM on overall and domain-specific cognitive impairment; and (3) whether CMM mediate the association between peripheral or auditory processing and cognitive function.

## Materials and methods

### Study design

The participants were volunteer members of the Shanghai study of health promotion for elderly individuals with frailty, which began in 2018 (Ruan et al., 2020a). For this study, potential participants were screened using face-to-face interviews, clinical examinations, laboratory tests, and neuropsychological testing, as previously reported (Ruan et al., 2020a; Ruan et al., 2020b; Ruan et al., 2020c). Informed consent was obtained from each volunteer or authorized using procedures approved by the Ethics Committee of Huadong Hospital (Approval No. Ref 2018K097 and No. Ref 2022K108).

A total of 801 participants were enrolled and evaluated during a comprehensive geriatric assessment visit at the Shanghai Key Laboratory of Clinical Geriatrics of Huadong Hospital, Fudan University. The 293 participants were excluded due to incomplete data for cognitive or hearing assessment or for the following reasons: (1) inadequate peripheral auditory function, e.g., an increase in the pure-tone threshold average (PTA) of 0.5, 1.0, or 2.0 kHz caused by middle and external ear disorders; (2) a word recognition score of less than 70% for the better ear in a quiet environment; (3) dementia that was clinically diagnosed according to the Diagnostic and Statistical Manual of Mental Disorders, Fourth Edition criteria (Cong et al., 2023); (4) severe disability and complete loss of vision; (5) acute inflammative diseases and traumatic brain injury, and stroke within 3 months; (6) psychiatric disorders and dyslexia; (7) patients using central nervous system medications that affect cognitive performance. The remaining 508 eligible participants were subsequently included in the present analysis. The research included 20 independent variables, the minimum sample size should be 10 to 15 times the number of independent variables (200–300 participants) based on Kendall’s sample size estimation method. To account for an approximate 15% rate of invalid or incomplete responses, the required sample size for this study was calculated to be 230–345 participants. A total of 508 valid responses and 392 valid responses in the sensitivity test exceeded the required threshold range, which could ensure sufficient statistical power for analysis.

### Hearing threshold and CAPD assessment

After the otoscope examination, all participants were asked to exclude middle and external ear disorders resulting in conductive hearing loss. PTA was performed with an audiometer (Conera Audiometer, GN Otometrics Ltd., Denmark) and a supra-aural earphone (TDH-39). Bilateral air- and bone-conduction thresholds were measured at 0.25, 0.5, 1, 2, 4, 6, and 8 kHz using standard audiometric assessment conducted by a certified audiologist at a sound-attenuating booth at the audiology center of Otolaryngology in Huadong Hospital. The air conduction PTAs of low frequencies (0.5, 1, and 2 kHz) (LPTA) and high frequencies (4, 6, and 8 kHz) (HPTA) were calculated separately. The best air-conduction LPTA from the left or right ears with normal tympanograms was used for analysis as a continuous variable. ARHL was defined as an LPTA threshold greater than 40 dB HL (hearing level) in the better ear, according to the World Health Organization definition of disabling hearing loss (World Health Organization, 1991). Tinnitus was assessed in those with self-reported chronic tinnitus (more than 3 months) using the tinnitus handicap inventory (THI). The THI is validated in Chinese people with a 25-item self-rating instrument and can yield a score that ranges from 0 to 100. Self-ratings of 0, 2, or 4 correspond to “not affected,” “sometimes affected,” and “always affected,” respectively.

A words-in-noise test was used to assess CAPD using a descending presentation paradigm following the WIN test procedure (Wilson et al., 2003; Wilson and Burks, 2005). Four standardized bisyllabic word lists (80 words total) were employed (Zhang et al., 2006), with two lists monaurally presented to each ear. To be included in this study, participants were required to demonstrate word recognition scores of 70% or higher at a suprathreshold of 40 dB HL or at a comfortable listening level in at least one ear in a quiet environment, with speech-shaped noise masking of the nontest ear. The word stimuli, spoken by a male talker in Mandarin, were presented against long-term average speech-spectrum noise generated from the test speech materials using Praat (v.5.1). Five unique words were presented at each of eight signal-to-noise ratios (SNRs), ranging from 24 dB to −4 dB S/N in 4 dB decrements. The presentation intensity of the speech stimuli was adjusted to each participant’s individualized comfortable level (approximately 40 decibel sensation level), whereas the background noise was incrementally varied in a progressive manner. The highest presentation level of the speech signal may reach 120 dB HL. The −4 dB S/N condition was included to minimize potential ceiling effects. Testing was conducted in a sound-attenuated booth using recorded materials delivered through a Madsen^®^ Astera2 audiometer and TDH-39 headphones at a comfortable listening level. If the average interaural hearing threshold difference exceeds 40 dB, speech-shaped noise is used to mask the better ear. The participants were instructed to repeat aloud each bisyllabic word they heard and were instructed to guess if uncertain. Correct repetitions were considered correct responses. The SNR corresponding to 50% correct word recognition was calculated using the Spearman–Kärber method (Finney, 1951) and was reported as the SNR threshold. Only those who could finish the neuropsychological assessments performed this task. Moreover, only the SNR from the better ear based on the LPTA was used to analyze the association between CAPD and cognitive performance in this study. For a dose–response analysis, we stratified the LPTA, HPTA, and SNR values of the better ear based on the LPTA into 3 tertiles based on performance strata.

### Cognitive function evaluation

A comprehensive neuropsychological test battery was administered during the geriatric comprehensive assessment visit at the Shanghai Key Laboratory of Clinical Geriatrics (2018–2024). Multiple standardized tests from several domains were administered: Trail Making Tests A and B (TMT A and B) for the executive/processing speed or attention domain; Boston Naming Test (BNT) and Animal List generation for the language domain; the Hopkins Verbal Learning Test-Revised (HVLT-R) for the memory domain, including delayed recall and recognition; and three process scores from the HVLT-R for identifying early pre-MCI. Words were presented binaurally at a comfortable level to ensure that each participant could listen clearly. To facilitate effect estimate comparisons across cognitive domains, domain- and process-specific *z* scores were calculated on the basis of a cognitive test categorization and previous work in this cohort (Ruan et al., 2020a; Ruan et al., 2020b; Ruan et al., 2020c). The corresponding normative *z* scores for neuropsychological tests could be calculated based on the raw score, predicted population mean score, and root mean square error of the regression equation (Ruan et al., 2020a). In addition to the Chinese version of the Mini-Mental State Examination (MMSE), global cognitive performance was also assessed using the normative *z* scores of three domains (two tests for each domain) and three learning processes of the Hopkins Verbal Learning Test-Revised (HVLT-R). If a participant had *z* scores > 1 standard deviation (SD) from the norm on TMT A or TMT B and intrusion errors or *z* scores < 1 SD from the norm on other tests of six batteries, the individual was defined as having an impaired total score or process score. Two impaired process scores, one impaired process score and one impaired total score, and an impaired total score across different cognitive domains were classified as pre-MCI (Thomas et al., 2018; Thomas et al., 2020; Ruan et al., 2021; Zhang et al., 2022). An impaired total score on two measures in the same domain and one impaired score in each of the three cognitive domains were classified as MCI.

### Clinical variables

Other variables collected from the study participants included demographic features (e.g., age, sex, and education level), body mass index (BMI), lifestyle factors (e.g., smoking, alcohol consumption), medical history of CMDs, and non-skin malignancy. CMDs were defined as type 2 diabetes, cardiovascular disease (heart disease and/or hypertension), and stroke (Luo et al., 2022; Tai et al., 2022; Dove et al., 2023; Li Q. Y. et al., 2024; Li H. et al., 2024). Heart disease includes coronary heart disease, myocardial infarction, angina or heart failure. Depressive symptoms were assessed using the 15-item Geriatric Depression Scale (GDS-15). Self-reported severity scores based on a brief version of the Neuropsychiatric Inventory Questionnaire (NPI-Q) were used to evaluate the severity of neuropsychiatric symptoms. Social dysfunction was assessed by using the 21-item Social Dysfunction Rating Scale. Physical frailty was evaluated using the five-item Fried scale with Chinese reference values with scores ranging from 0 to 5 (with scores 3–5 and 1–2 indicating frailty and prefrailty, respectively). Assessments of these covariates have been described in detail previously (Ruan et al., 2020a; Ruan et al., 2020b; Zhang et al., 2021).

### Statistical analysis

**Figure 1:**
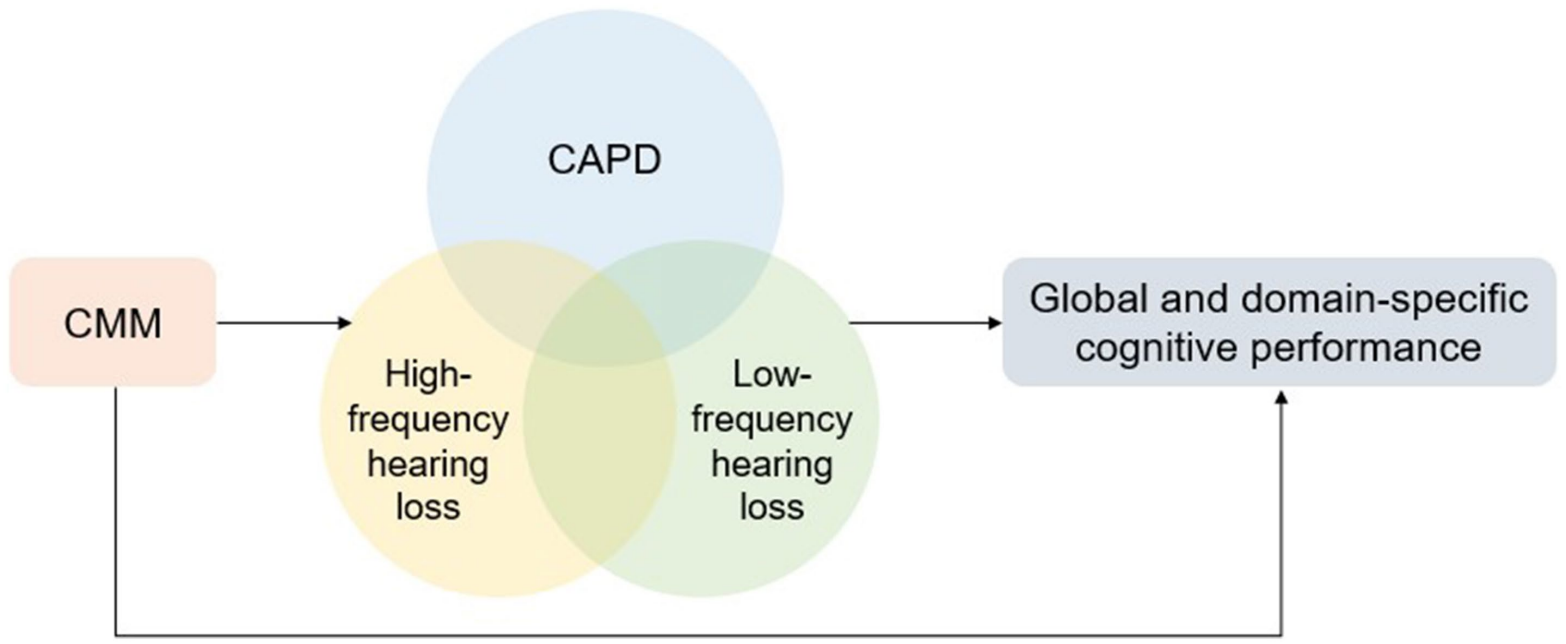
The hypothesized causal pathway for CMM mediating the association between low- or high-frequency hearing loss, or CAPD, and global or domain specific cognitive performance.

In the study, we hypothesized that both age-related peripheral or central hearing loss, and CMM are independently associated with global or domain-specific cognitive impairment. Moreover, CMM might indirectly affect cognitive performance by mediating the association between age-related peripheral or central hearing loss and cognitive impairment (Figure 1). Demographic characteristics, CMDs, thresholds of LPTA, HPTA, SNRs from the better ear, and additional covariates were compared across global cognitive status categories. Continuous variables were analyzed using the Wilcoxon test due to non-normality, whereas categorical variables were assessed using the chi-square tests. Qualitative variables are summarized as frequencies and percentages, and quantitative variables are summarized as the means and standard deviations. The maximum data missing for covariant (social dysfunction) was less than 7.5% in total sample, and 5.51% in sensitivity test. Bonferroni method was used to correct *p*-values for multiple comparisons. Multivariate logistic regression models were employed to evaluate associations between global cognitive performance or domain-specific impairments (e.g., processing speed/attention, executive function, memory, language) and CMDs, the values of LPTA, HPTA, and SNR. To assess the links between SNR value and global or domain-specific cognitive impairment, Model 1 was adjusted for LPTAs, HPTAs, and the demographic factors of age, sex, and education. Model 2 included BMI, scores, non-skin malignancy, CMDs, frailty status, tinnitus THI, GDS, social dysfunction, and NPI scores. To assess the associations between LPTAs and global or domain-specific cognition performance, the LPTAs and HPTAs in Model 1 were replaced with HPTAs and SNRs. For the assessment of the association between HPTAs and global or domain-specific cognition performance, the LPTAs and HPTAs in Model 1 were replaced with LPTA and SNRs. To assess the associations between CMM and global or domain-specific cognitive impairment, Model 1 was adjusted for LPTAs, HPTAs, SNRs, and the demographic factors of age, sex, education, and CMDs were excluded from Model 2.

To assess the independent and joint effects of the cumulative numbers of CMDs and LPTAs, HPTAs or SNRs on cognitive performance, weighted logistic regression were used. Participants were stratified into tertiles on the basis of the values of LPTA, HPTA or SNR and grouped by CMD count (0–3). Mediation analyses, performed using the R package “Mediation,” were used to examine whether CMDs mediated the relationship between peripheral/central auditory dysfunction and cognitive outcomes. All the statistical procedures were executed in R software (version 4.2). Adjusted *p*-values by false discovery rate method was used to account for multiple comparisons in all models. We emphasized that the effects and mediation refer to statistical and exploratory, rather than causal associations.

### Sensitivity analysis

We performed a sensitivity analysis in which we reran the main analysis using an LPTA cutoff value of > 40 dB HL. This exclusion may reduce the effect of audibility on CAPD and assess the robustness of the association between increased LPTA, HPTA or CAPD and cognitive impairment. The cutoff value (Sardone et al., 2020; Sardone et al., 2021), or PTA cutoff value > 35 dB HL (Cooper and Gates, 1991; Gates et al., 2002) had been widely used to assess the effect of CAPD on cognitive performance.

## Results

### Characteristics of the study population

The clinical and sociodemographic characteristics of the study population, which were divided into three groups according to global cognitive function, are shown in Table 1. Compared with cognitively normal participants, individuals with pre-MCI and MCI had a significantly higher percentage of low education levels, CMDs, impairments in global and specific cognitive domains, SNR values, NPI scores, and severe frailty. Individuals with pre-MCI included significantly fewer men; had a lower percentage of non-skin malignancies; used less alcohol. Individuals with MCI were significantly older and high numbers of CMM; had depressive and self-reported neuropsychiatric symptoms; and had other dysfunctions, including social function, GDS scores, LPTA, and HPTA values. Compared with those with pre-MCI, those with MCI had more severe global cognitive impairment, low- and high-frequency hearing loss, and CAPD (Table 1). However, after adjusted *p*-value using the Bonferroni method, the significant difference could only be observed in the percentages of cardiovascular disease, stroke, and executive, memory, and language impairments, and MMSE score in the pre-MCI vs. the cognitive normal groups (Supplementary Table 1). The significant difference in the MCI vs. the cognitively normal groups, included the percentages of cardiovascular disease, education levels, and executive, memory, and language impairments, MMSE score, and the values of LPTA, HPTA, and SNR. The results remained consistent after excluding those with LPTA > 40 dB HL (Supplementary Table 2-1; Supplementary Table 2-2).

**Table 1:** Characteristics of the study population by cognitive status (*n* = 509).

Characteristic	Cognitively normal control (*n* = 193)	Pre-MCI (*n* = 144)	MCI (*n* = 171)	*p* (PreMCI vs. control)	*p* (MCI vs. control)	*p* (PreMCI vs. MCI)
Age (years)	72.00 (68.00–77.00)	73.00 (69.00–78.00)	73.00 (69.00–78.75)	0.106	0.022	0.511
Male sex, %	98 (50.78)	53 (36.81)	75 (43.86)	0.015	0.225	1.000
Education				0.075	1.152e-05	1.000
Illiterate or primary education, %	2 (1.04)	7 (4.86)	18 (10.53)			
Middle school education, %	111 (57.51)	87 (60.42)	111 (64.91)			
College education or more, %	77 (39.90)	50 (34.72)	41 (23.98)			
Cardiovascular disease, %	95 (49.22)	99 (68.75)	116 (67.84)	4.481e-04	5.989e-04	1.000
Diabetes, %	28 (14.51)	31 (21.53)	44 (25.73)	0.129	0.0123	1.000
Stroke, %	13 (6.74)	9 (6.25)	24 (14.04)	5.616e-23	0.0364	1.000
Non-skin malignancy, %	28 (14.51)	8 (5.56)	17 (9.94)	0.0137	0.231	1.000
The number of cardometabolic multimorbidity	2.00 (1.00–3.00)	2.00 (1.00–3.00)	2.00 (1.00–3.00)	0.126	0.014	0.429
BMI	24.01 (21.67–25.80)	24.02 (21.49–25.87)	24.22 (22.00–26.10)	0.909	0.424	0.450
Smoking, %				0.097	0.783	1.000
No smoking	140 (72.54)	121 (84.03)	136 (79.53)			
Former	22 (11.40)	8 (5.56)	17 (9.94)			
Current	15 (7.77)	9 (6.25)	13 (7.60)			
Alcohol use, %				0.041	0.525	1.000
No alcohol	150 (77.72)	129 (89.58)	143 (83.63)			
Former	15 (7.77)	5 (3.47)	9 (5.26)			
Current	13 (6.74)	4 (2.78)	14 (8.19)			
Living alone, %	14.00 (7.25)	14.00 (9.72)	15.00 (8.77)	0.535	0.793	1.000
Social dysfunction score	27.00 (23.00–32.00)	28.00 (23.00–34.00)	28.00 (24.00–36.00)	0.458	0.0480	0.221
GDS15 score	3.00 (1.00–5.00)	3.00 (1.50–5.00)	3.00 (2.00–5.00)	0.598	0.050	0.194
MMSE score	28.00 (27.00–29.00)	27.00 (26.00–28.00)	26.00 (25.00–28.00)	1.359e-05	5.505e-13	7.69e-04
NPI score	0.00 (0.00–2.00)	1.00 (0.00–3.00)	1.00 (0.00–3.00)	0.035	0.016	0.819
THI score	0.00 (0.00–24.00)	2.00 (0.00–26.00)	0.00 (0.00–22.00)	0.632	0.432	0.231
Frailty score	1.00 (0.00–1.00)	1.00 (0.00–2.00)	1.00 (0.00–2.00)	0.0515	0.0021	0.291
Low_Frq PTA	25.00 (18.30–35.00)	28.30 (21.70–38.00)	31.70 (25.00–45.00)	0.117	9.89e-06	0.0035
High_Frq PTA	47.50 (32.50–60.00)	50.00 (36.70–62.50)	55.00 (40.00–65.00)	0.158	1.90e-04	0.024
SNR	−4.40 (−5.20- -2.80)	−3.60 (−5.20- -1.20)	−2.80 (−4.40–0.40)	0.049	3.310e-06	0.019
Attention/executive domain decline (TMT A & B), %	10 (5.18)	45 (31.25)	107 (62.57)	3.924e-10	4.691e-31	1.000
Memory domain decline (delayed recall & recognition), %	45 (23.32)	91 (63.19)	144 (84.21)	3.614e-13	1.317e-30	1.000
Language domain decline (BNT & animal fluency test), %	19 (9.84)	67 (46.53)	118 (69.01)	5.699e-14	1.055e-30	1.000

Low_Frq, low-frequency; High_Frq, high-frequency; BMI, body mass index; MMSE, the Mini-Mental Status Exam; MCI, mild cognitive impairment; GDS, the Geriatric Depression Scale; NPI, self-report Neuropsychiatric Inventory Questionnaire; PTA, pure tone average; SNR, signal-to-noise ratio; SD, standard deviation; THI, Tinnitus Handicap Inventory.

### Associations between CMM, LPTA, HPTA, or SNR and global and domain-specific cognitive performance

The associations between LPTA, HPTA, SNR, and CMM and global cognitive performance among the three groups across all the samples and the sensitivity test results are shown in Model 1 (Supplementary Table 3). As a continuous or categorical variable, CMM was significantly associated with global cognitive performance between the cognitively normal and pre-MCI groups and between the cognitively normal and MCI groups but not between the pre-MCI and MCI groups. The significant association between the LPTA, the HPTA, or SNR values and global cognition were observed between the cognitively normal group and the MCI group in the total sample and the sensitivity test. In addition, the LPTA value was significantly associated with global cognition between the pre-MCI and MCI groups in the total sample; the HPTA value was marginally associated with global cognition in the sensitivity test between the cognitively normal group and the pre-MCI group.

In Model 2, a significant association between CMM and global cognitive performance was still detected between the cognitively normal group and the pre-MCI group (adjusted *p* = 0.0068 in total sample, and *p* = 0.0506 in sensitivity test) and between the cognitively normal group and the MCI group (adjusted *p* = 0.0068 in total sample, and *p* = 0.012 in sensitivity test) (Table 2). Significant associations between the SNR value and global cognition between the cognitively normal group and the MCI group (adjusted *p* = 0.044 in total sample, and *p* = 0.051 in sensitivity test), and no association between the cognitively normal group vs. the pre-MCI group, or the cognitively normal group vs. the MCI group was detected. No significant association between LPTA, or HPTA and global cognition was observed in three comparison groups.

**Table 2:** Association of overall cognitive performance with hearing loss of low- and high-frequency PTA, and SNR in the better ear, or CMM.

Comparison of groups	Variable	Total sample test Model 2			Sensitivity test Model 2		
		*β* (95% CI)	*p*-value	Adjusted *p*-value	*β* (95% CI)	*p*-value	Adjusted *p*-value
Pre-MCI vs. cognitively intact	Low_Frq PTA	−0.003 (−0.009, 0.003)	0.314	0.435	0.000 (−0.010, 0.009)	0.931	0.931
	High_Frq PTA	0.004 (−0.001, 0.009)	0.103	0.185	0.003 (−0.002, 0.009)	0.224	0.310
	SNR	0.017 (0.000, 0.034)	0.056	0.112	0.022 (−0.003, 0.046)	0.081	0.208
	CMD (continuous)	0.124 (0.047, 0.202)	0.0019	0.0068	0.121 (0.032, 0.209)	8.435e-03	0.0506
	CMD = 0	Ref			Ref		
	CMD = 1	0.130 (0.005, 0.254)	0.0429	0.097	0.110 (−0.030, 0.250)	0.124	0.248
	CMD ≥ 2	0.273 (0.107, 0.439)	0.0015	0.007	0.243 (0.046, 0.440)	0.017	0.051
MCI vs. cognitively intact	Low_Frq PTA	0.004 (−0.001, 0.010)	0.121	0.198	0.003 (−0.006, 0.012)	0.494	0.593
	High_Frq PTA	0.002 (−0.002, 0.006)	0.421	0.539	0.004 (−0.001, 0.009)	0.108	0.243
	SNR	0.019 (0.004, 0.034)	0.0148	0.044	0.026 (0.005, 0.047)	0.0163	0.051
	CMD (continuous)	0.131 (0.055, 0.206)	8.29e-04	0.0068	0.151 (0.065, 0.237)	6.650e-04	0.012
	CMD = 0	Ref			Ref		
	CMD = 1	0.193 (0.074, 0.311)	1.59e-03	0.0068	0.166 (0.032, 0.301)	0.0165	0.051
	CMD ≥ 2	0.276 (0.117, 0.436)	8.73e-04	0.0068	0.296 (0.118, 0.474)	1.515e-03	0.014
MCI vs. pre-MCI	Low_Frq PTA	0.006 (0.001, 0.012)	0.0319	0.082	0.003 (−0.007, 0.014)	0.539	0.606
	High_Frq PTA	−0.001 (−0.006, 0.004)	0.726	0.751	0.001 (−0.004, 0.007)	0.684	0.724
	SNR	0.003 (−0.014, 0.019)	0.751	0.751	0.012 (−0.012, 0.035)	0.333	0.428
	CMD (continuous)	0.032 (−0.055, 0.118)	0.475	0.539	0.070 (−0.030, 0.170)	0.169	0.304
	CMD = 0	Ref			Ref		0.308
	CMD = 1	0.106 (−0.044, 0.257)	0.168	0.252	0.117 (−0.057, 0.292)	0.188	
	CMD ≥ 2	0.069 (−0.121, 0.259)	0.479	0.539	0.143 (−0.078, 0.364)	0.207	0.310

CMD, cardiometabolic disease; CI, confidence interval; Low_Frq, low-frequency; High_Frq, high-frequency; MCI, mild cognitive impairment; PTA, pure tone average; SNR, signal-to-noise ratio. Adjusted *p*-value was corrected by using false discovery rate method.

CMM was independently associated with attention/executive function (by TMT B) and language (by animal fluency) in the total sample and sensitivity test in Model 1 (Supplementary Table 4). LPTA was independently associated with delayed recall in the total sample and sensitivity test. HPTA was independently associated with processing speed and delayed recall in the total sample, attention/executive function in the sensitivity test. The SNR was independently associated with delayed recall in the total sample and sensitivity test.

CMM was still independently associated with attention/executive function in the sensitivity test (*β*, 0.087; 95% CI, 0.028, 0.145; adjusted *p* = 0.035) in Model 2 (Table 3). HPTA remained independently associated with attention/executive function in the sensitivity test (*β*, 0.005; 95% CI, 0.001, 0.008; adjusted *p* = 0.0395) (Table 3). No significant association between LPTA, or SNR and domain-specific cognitive performance was detected after *p* correction.

**Table 3:** Association of domain-specific cognitive performance with hearing loss of low- and high- frequency PTA, and SNR in the better ear, or CMM.

Domain	Variables	Total sample Model 2			Sensitivity test Model 2		
		*β* (95% CI)	*p*-value	Adjusted *p*-value	*β* (95% CI)	*p*-value	Adjusted *p*-value
TMT A	Low_Frq PTA	0.001 (−0.003, 0.005)	0.660	0.742	0.001 (−0.005, 0.007)	0.732	0.941
	High_Frq PTA	0.002 (−0.001, 0.005)	0.270	0.405	0.003 (−0.001, 0.006)	0.112	0.366
	SNR	0.002 (−0.009, 0.013)	0.703	0.742	0.000 (−0.015, 0.015)	0.998	0.998
	CMM (continuous)	0.042 (−0.013, 0.096)	0.134	0.329	0.032 (−0.027, 0.091)	0.294	0.557
	CMM = 0	Ref			Ref		
	CMM = 1	0.019 (−0.066, 0.104)	0.660	0.742	−0.022 (−0.111, 0.068)	0.636	0.881
	CMM ≥ 2	0.123 (0.006, 0.240)	0.041	0.246	0.082 (−0.049, 0.214)	0.221	0.510
TMT B	Low_Frq PTA	0.000 (−0.004, 0.004)	0.987	0.987	−0.004 (−0.010, 0.003)	0.241	0.510
	High_Frq PTA	0.003 (0.000, 0.006)	0.0868	0.298	0.005 (0.001, 0.008)	5.493e-03	0.0395
	SNR	−0.003 (−0.003, −0.013)	0.635	0.742	0.000 (−0.015, 0.014)	0.953	0.980
	CMM (continuous)	0.068 (0.015, 0.121)	0.012	0.108	0.087 (0.028, 0.145)	3.850e-03	0.035
	CMM = 0	Ref			Ref		
	CMM = 1	0.125 (0.042, 0.208)	3.238e-03	0.058	0.164 (0.076, 0.252)	3.230e-04	0.0078
	CMM ≥ 2	0.142 (0.041, 0.242)	6.267e-03	0.075	0.194 (0.088, 0.300)	4.360e-04	0.0078
Delayed recall	Low_Frq PTA	0.005 (0.001, 0.009)	0.0157	0.113	0.007 (0.000, 0.014)	0.0396	0.204
	High_Frq PTA	−0.001 (−0.004 0.002)	0.608	0.742	−0.001 (−0.004 0.003)	0.714	0.941
	SNR	0.010 (−0.001, 0.021)	0.084	0.298	0.015 (−0.001, 0.030)	0.064	0.263
	CMM (continuous)	0.036 (−0.019, 0.091)	0.205	0.388	0.035 (−0.027, 0.097)	0.270	0.54
	CMM = 0	Ref			Ref		
	CMM = 1	0.060 (−0.028, 0.148)	0.179	0.379	0.036 (−0.061, 0.133)	0.466	0.763
	CMM ≥ 2	0.074 (−0.045, 0.192)	0.223	0.401	0.083 (−0.053, 0.218)	0.233	0.510
Recognition	Low_Frq PTA	−0.003 (−0.007, 0.002)	0.264	0.405	0.000 (−0.008, 0.007)	0.919	0.973
	High_Frq PTA	0.003 (−0.007, 0.002)	0.264	0.405	0.002 (−0.003, 0.006)	0.442	0.758
	SNR	0.011 (−0.002, 0.023)	0.091	0.298	0.015 (−0.003, 0.033)	0.0944	0.309
	CMM (continuous)	0.022 (−0.040, 0.084)	0.483	0.669	0.010 (−0.062, 0.081)	0.790	0.963
	CMM = 0	Ref			Ref		
	CMM = 1	0.071 (−0.028, 0.169)	0.162	0.365	0.028 (−0.086, 0.141)	0.630	0.881
	CMM ≥ 2	0.040 (−0.095, 0.176)	0.559	0.742	0.015 (−0.139, 0.170)	0.848	0.963
BNT	Low_Frq PTA	0.004 (0.000, 0.008)	0.0529	0.272	0.002 (−0.005, 0.009)	0.490	0.767
	High_Frq PTA	−0.002 (−0.005, 0.002)	0.327	0.471	0.000 (−0.004, 0.003)	0.856	0.963
	SNR	0.009 (−0.003, 0.020)	0.137	0.329	0.004 (−0.012, 0.021)	0.623	0.881
	CMM (continuous)	0.047 (−0.010, 0.104)	0.108	0.302	0.062 (−0.004, 0.127)	0.066	0.263
	CMM = 0	Ref			Ref		
	CMM = 1	0.017 (−0.074, 0.107)	0.721	0.742	0.012 (−0.091, 0.115)	0.814	0.963
	CMM ≥ 2	0.069 (−0.053, 0.190)	0.269	0.405	0.102 (−0.039, 0.242)	0.157	0.404
Animal fluency	Low_Frq PTA	0.004 (−0.001, 0.008)	0.109	0.302	0.001 (−0.007, 0.008)	0.887	0.968
	High_Frq PTA	−0.001 (−0.004, 0.003)	0.6996	0.742	0.002 (−0.002, 0.006)	0.372	0.670
	SNR	−0.003 (−0.015, 0.009)	0.658	0.742	−0.016 (−0.033, 0.001)	0.073	0.263
	CMM (Continuous)	0.057 (−0.003, 0.117)	0.065	0.293	0.079 (0.011, 0.147)	0.023	0.138
	CMM = 0	Ref			Ref		
	CMM = 1	0.147 (0.051, 0.243)	2.955e-03	0.058	0.159 (0.052, 0.266)	3.834e-03	0.035
	CMM ≥ 2	0.079 (−0.043, 0.200)	0.205	0.388	0.108 (−0.029, 0.244)	0.124	0.343

CMD, cardiometabolic disease; CI, confidence interval; Low_Frq, low-frequency; High_Frq, high-frequency; PTA, pure tone average; SNR, signal-to-noise ratio; HVLT-R, the Hopkins Verbal Learning Test-Revised; BNT, Boston Naming Test; TMT Part A and B, Trail Making Test A and B. Adjusted *p*-value was corrected by using false discovery rate method.

### The joint effects of CMM and LPTA, HPTA, or the SNR on global and domain-specific cognitive performance

The dose–response relationships of CMM and LPTA, HPTA, or SNR value on global cognition were observed among three groups in Model 1 (Supplementary Table 5). In the cognitively normal vs. pre-MCI groups, the significant joint effects of LPTA and CMM on global cognition were detected in individuals with CMD ≥ 2, tertile 1 in the total sample, and CMD ≥ 2, tertile 2 in the total sample and the sensitivity test. The significant joint effects of HPTA and CMM on global cognition were detected in individuals with CMD ≥ 2, tertile 2 in total sample and the sensitivity test. The significant joint effects of SNR and CMM on global cognition were detected in individuals with CMD ≥ 2, tertile = 1 and CMD = 1, tertile 3 in the total sample. In the cognitively normal vs. MCI groups, the significant joint effects of LPTA and CMM on global cognition were detected in individuals with CMD ≥ 2, tertile 1; CMD = 1 or ≥ 2, tertile 2; CMD = 1 or ≥ 2, tertile 3 in total sample and the sensitivity test, as well as CMD = 0, tertile 3 in the total sample. The significant joint effects of HPTA and CMM on global cognition were detected in individuals with CMD = 1 or ≥ 2, tertile 3 in the total sample and the sensitivity test, and CMD = 1, tertile 2 in the total sample, as well as CMD ≥ 2, tertile 2 in the sensitivity test. The significant joint effects of SNR and CMM on global cognition were detected in individuals with CMD = 1 or ≥ 2, tertile 3 in the total sample and the sensitivity test, and CMD = 1, tertile 1 or 2; CMD ≥ 2, tertile 1 or 2 in the total sample. In the pre-MCI vs. MCI groups, the significant joint effect of LPTA and CMM on global cognition was detected in individuals with CMD = 1, tertile 3 in total sample. The significant joint effect of HPTA and CMM on global cognition was detected in individuals with CMD ≥ 2, tertile 3 in total sample. No significant joint effect of SNR and CMM on global cognition.

**Figure 2:**
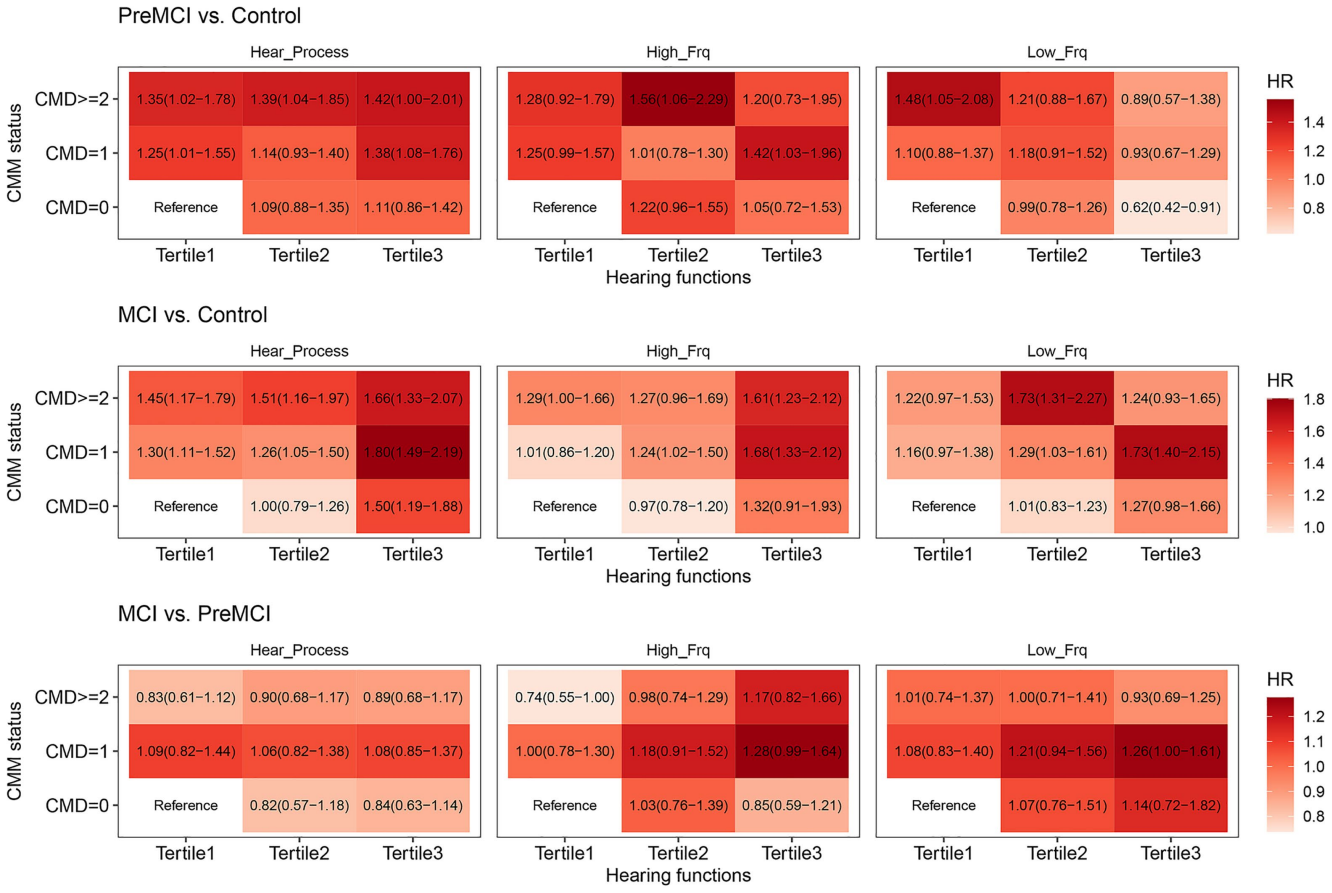
The joint effect of CMM and LPTA, HPTA, or CAPD on the overall cognitive function in the total sample.

**Figure 3:**
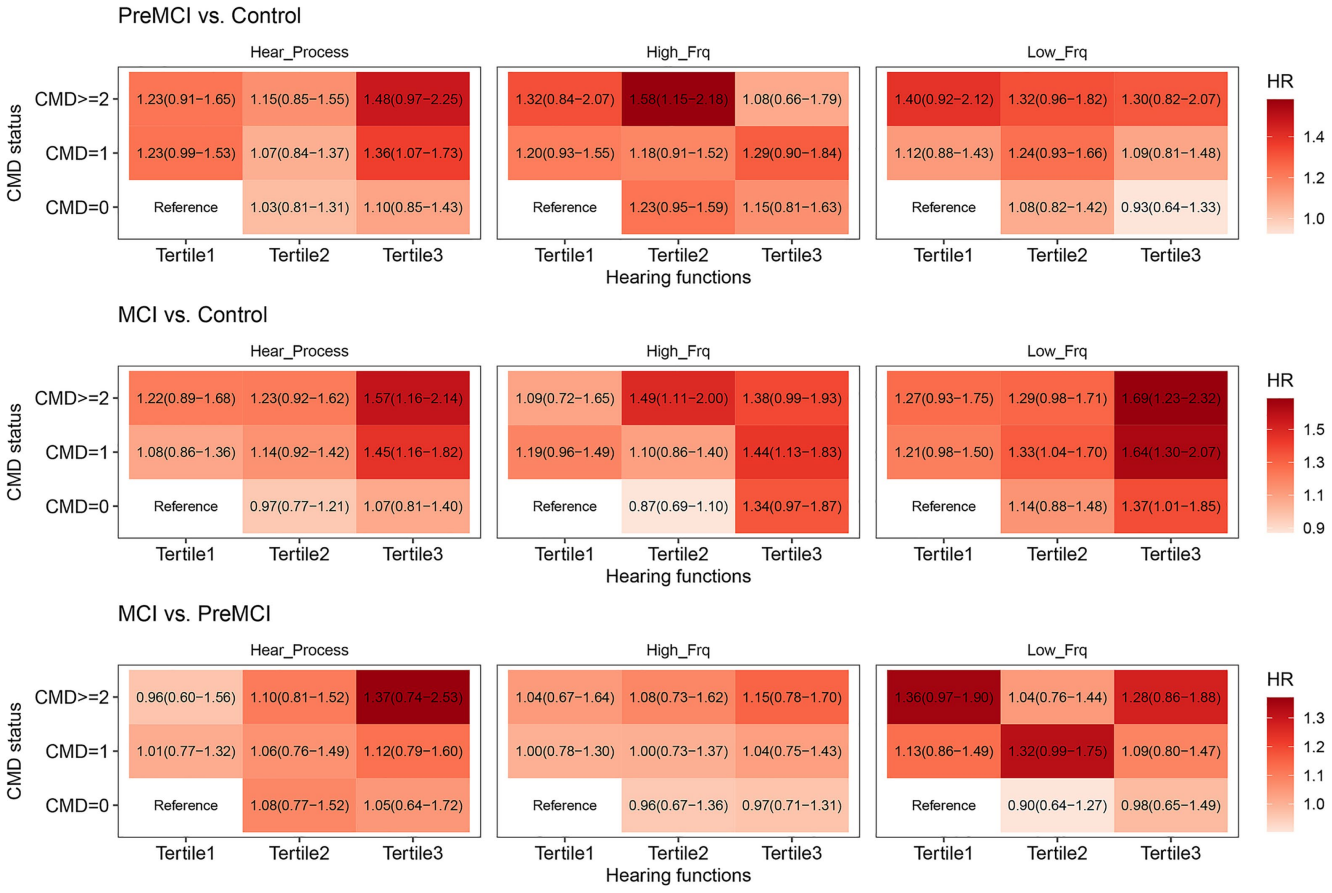
The joint effect of CMM and LPTA, HPTA, or CAPD on the overall cognitive function in the sensitivity test.

After *p*-value correction in Model 2, the significant joint effect of LPTA, HPTA, or SNR and CMM on global cognition still could be detected in the cognitively normal vs. the pre-MCI groups (Figures 2, 3). Individuals with CMD ≥ 2, tertile 2 (adjusted *p* = 0.0043), and CMD = 1, tertile 3 (adjusted *p* = 9.975e-05) in the total sample, and CMD = 1, tertile 3 (adjusted *p* = 0.008), and CMD ≥ 2, tertile 3 (*p* = 0.067) in the sensitivity test, presented significant/marginal joint effects of LPTA and CMM on global cognition. Individuals with CMD = 1, tertile 3 (adjusted *p* = 5.983e-04), and CMD ≥ 2, tertile 3 (adjusted *p* = 0.011) in total sample, and CMD = 1, tertile 3 in the sensitivity test, presented significant joint effects of HPTA and CMM on global cognition. These with CMD = 1, tertile 1 (adjusted *p* = 0.0140), CMD ≥ 2, tertile 1 (adjusted *p* = 0.011), CMD ≥ 2, tertile 2 (adjusted *p* = 0.021), CMD = 0, tertile3 (adjusted *p* = 0.0107), CMD = 1, tertile 3 (adjusted *p* = 3.023e-06), and CMD ≥ 2, tertile 3 (adjusted *p* = 5.983e-04) in total sample, and CMD = 1, tretile 3 (adjusted *p* = 0.0579) in the sensitivity test, presented significant/marginal joint effects of SNR and CMM on global cognition.

The dose–response relationships of CMM and LPTA, HPTA, or SNR value on specific cognitive domain in Model 1 were showed in Supplementary Table 6. The significant dose–response relationships of the increase in the cumulative number of CMD and LPTA on executive function were detected in those with CMD = 1, tertile 1 or 2; CMD ≥ 2, tertile 2 in total sample, and CMD ≥ 2, tertile 3 in the sensitivity test. The significant jointeffects of LPTA and CMM on language by BNT also was detected in these with CMD, ≥ 2, tertile 2 and CMD = 0, tertile 3 in the total sample; as well as language by animal fluency in these with CMD = 1, tertile 2 in total sample and the sensitivity, CMD = 1, tertile 1 and CMD ≥ 2, tertile 2 in the total sample. The significant jointeffects of HPTA and CMM on processing speed were detected in these with CMD ≥ 2, tertile 3 in the sample and the sensitivity test; executive function in these with CMD = 1 or ≥ 2, tertile 3 in the total sample and the sensitivity test, CMD = 1, tertile 2 in total sample; as well as language by animal fluency in these with CMD = 1, tertile 2 in the total sample. The significant jointeffects of SNR and CMM on executive function were detected in these with CMD ≥ 2, tertile 3 in the total sample and the sensitivity test, CMD = 1, tertile 1; and CMD = 1 or ≥ 2, tertile 2 in total sample; delayed memory in these with CMD = 1 or ≥ 2, tertile 3 in the total sample; and language by animal fluency in these with CMD = 1 or ≥ 2, tertile 1 in the total sample.

In Model 2, no significant dose–response relationships of CMM and LPTA or SNR on domain-specific cognitive performance after adjusted for all confounders and *p*-value correction (Supplementary Table 7). The significant joint effect of CMM and HPTA on executive function was only detected in these with CMD ≥ 2, tertile 3, and marginal effect in these with CMD = 1, tertile 3 in sensitivity test (adjusted *p* = 0.0775).

### CMM mediated the associations between the LPTA, HPTA, or SNR and global and domain-specific cognitive performance

Mediation analysis of CMM for the total sample on the association between LPTA, HPTA, or SNR, and global or domain-specific cognitive performance after adjustment for all confounders in Model 2 revealed that no significant mediation effect was observed (Supplementary Tables 8, 9). However, in the sensitivity test in Model 2, CMM significantly mediated the effects of HPTA on global and domain-specific (processing speed and attention/executive function) cognitive performance. The average direct effect (ADE) [3.10e-03 (−2.599e-04, 4.666e-03); *p* = 0.064] of the HPTA, the average causal mediation effect (ACMD) [5.003e-04 (−9.797e-06, 1.385e-03); *p* = 0.062], and the mediation proportion [0.139 (−9.482e-03, 0.894); *p* = 0.074] of CMM on global cognition were marginally significant in the preMCI group vs. the cognitively normal group. The ADE [3.53e-03 (1.508e-03, 4.375e-03); *p* = 0.006] of the HPTA, the ACMD [6.809E-04 (9.675e-05, 1.623e-03); *p* = 0.010], and the mediation proportion [0.162 (0.026, 0.493); *p* = 0.010] of CMM on global cognition were significant in the MCI group vs. the cognitively normal group. The percentage of CMM that mediated the relationship between the HPTA and global cognitive performance was 16.172% (Supplementary Table 8). The ADE of HPTA on processing speed was [1.496e-03 (4.361e-04, 1.872e-03); *p* = 0.016], while the total effect was [1.665e-03 (7.407e-04, 2.026e-03); *p* = 0.008]. The indirect effect of CMM-mediated processing speed by HPTA was [1.683e-04 (4.098e-06, 5.051e-04); *p* = 0.044]. This indicated that approximately 10.10% of the total effect of HPTA on processing speed was explained by the mediated effect through CMM (*p* = 0.052). The ADE of HPTA on executive function was [1.378e-03 (5.013e-04, 1.764e-03); *p* = 0.024], while the total effect was [1.666e-03 (8.765e-04, 2.053e-03); *p* = 0.006]. The indirect effect of CMM-mediated executive function by HPTA was [1.683e-04 (4.098e-06, 5.051e-04); *p* = 0.018]. Overall, 17.30% of the total effect of HPTA on executive function was explained by the mediated effect through CMM (*p* = 0.024) (Supplementary Table 9 and Supplementary Figure 1).

After *p*-value was corrected by using false discovery rate method, the CMM still significantly mediated the effects of HPTA on global cognition in the MCI group vs. the cognitively normal group (Supplementary Table 9). The ADE (adjusted *p* = 0.050) of the HPTA, the ACMD (adjusted *p* = 0.050), the total effect (adjusted *p* < 0.0001) by CMM, and the mediation proportion (adjusted *p* = 0.050) of CMM on global cognition were significant.

## Discussion

We found that CMM was independently associated with global cognitive impairment (in the pre-MCI and MCI groups) and attention/executive function in the sensitivity test. HPTA was independently associated with attention/executive function in sensitivity test. CAPD was independently associated with global cognition in the cognitively normal and MCI groups. The dose–response relationships of CMM and LPTA, HPTA, or CAPD on global cognition could be seen in the cognitively normal vs. MCI groups. The dose–response relationship of CMM and HPTA on executive function was significant in the sensitivity test. In the sensitivity experiment, CMM significantly mediated the associations between the HPTA and global cognition in the cognitively normal and MCI groups.

Previous studies examined the independent associations between peripheral hearing loss, CAPD or individual CMDs and global or domain-specific cognitive performance. Several systematic reviews and meta-analyses have indicated that peripheral hearing loss is associated with MCI (Lau et al., 2022) and dementia (Loughrey et al., 2018) in cross-sectional studies. Population-based prospective cohort studies have shown that peripheral hearing loss significantly increases the risk of MCI (Lau et al., 2022; Yu et al., 2024), dementia (Ford et al., 2018; Yu et al., 2024), cognitive decline (Yu et al., 2024), and AD dementia (Liang et al., 2021; Yu et al., 2024). ARHL or ARHL severity is associated with domain-specific cognitive impairment, including low executive function, delayed memory, and language function (Lin et al., 2013; Armstrong et al., 2020; Zhang et al., 2022; Samelli et al., 2022). Our results showed that peripheral hearing loss was not significantly associated with global and domain-specific cognitive performance after adjusted for all confounders and *p*-value correction. Similar to our results in this study, CAPD was significantly associated with global cognition, and marginally associated with delayed recall after adjustment for all confounders. This conclusion is consistent with the sensitivity test in a recent study (Mamo et al., 2019). However, after *p*-value correction, CAPD was only significantly associated with global cognition. Previous studies have shown that CAPD is associated with memory impairment or AD (Gates et al., 2008; Idrizbegovic et al., 2011; Nixon et al., 2019), preclinical AD (Byun et al., 2023), MCI (Idrizbegovic et al., 2011; Jalaei et al., 2019; Sardone et al., 2020), and dementia (Nixon et al., 2019; Sardone et al., 2020). Some cognitive domains, such as processing speed, inhibitory control, episodic and working memory, and executive function, are associated with CAPD (Dryden et al., 2017; Nixon et al., 2019). A marginal association between pre-MCI and CAPD was observed in this study potentially because pre-MCI includes other domain impairments in addition to preclinical AD or memory impairment. Episodic long-term memory has been proposed for the automatic processing of speed in noise. The prefrontal cortex, which controls executive functioning and working memory, is important for the processing of rapid auditory signals from noise with increased cognitive effort. This effort further influences other cognitive domains (Ronnberg et al., 2013; Ronnberg et al., 2019).

LPTA and HPTA are associated with global cognitive performance in several studies (Mukari et al., 2017; Xu et al., 2021; Wang et al., 2022; Zhang et al., 2024) as well as domain-specific cognitive performance (Nicholas et al., 2021; Diao et al., 2021; Fu et al., 2021). However, after adjustment for all confounders in our study, only the significant association between LPTA and global cognition in pre-MCI vs. MCI groups, and between LPTA and delayed memory in both total sample or sensitivity test. Only the significant association between HPTA and attention/executive function was detected in the sensitivity test. A possible explanation for the difference with other studies is that the study subjects is the volunteers from different communities of Shanghai Municipality. In addition, hearing loss in speech frequency might more significantly affect audibility. However, after *p*-value correction, only HPTA remained significant association with executive function in the sensitivity test.

Recent studies with large samples have indicated that the presence of hypertension, CAD and diabetes is associated with poorer cognitive performance. An increasing number of CMDs is dose-dependently associated with a decline in the cognitive function score (Lyall et al., 2017; Jin et al., 2023). Longitudinal cohort studies have indicated that CMM is independently associated with the risk of dementia and extensive structural changes in the brain, including lower hippocampal and total gray matter volume (Tai et al., 2022). Individuals with CMM had greater dementia risk than individuals with single CMDs did, and individuals with mid-life CMDs had greater dementia risk than those with late-life CMDs did (Dove et al., 2023). Our results were consistent with those of previous studies. CMM was independently associated with pre-MCI and MCI after adjustment for all confounders and *p*-value correction. An increasing number of CMDs for increasing thresholds (severity of hearing loss, from tertiles 1 to 3) of LPTA, HPTA, or SNR was dose-dependently associated with an increase in the hazard ratio (HR) of cognitive impairment in the MCI group vs. the cognitively normal group. Moreover, our results also revealed a dose-dependent relationship between the severity of low- or high-frequency hearing loss, or CAPD, and the HR of cognitive impairment. In addition, CMM and LPTA, HPTA, or SNR had significant jointeffects on executive, language, or delayed memory functioning after adjusted for all confounders. But only the significant joint effect of CMM and HPTA on executive function after *p*-value correction. After the exclusion of audibility problems resulting from the increase in LPTA, CMM significantly mediated the association between the HPTA and global cognitive performance, and marginally mediated the association between HPTA and executive functioning (adjusted *p* = 0.092). The mediation proportions were 16.172 and 17.27%, respectively. However, the mediation effect sizes were very small and clinically unclear. Although HPTA was independently associated with executive function (adjusted *p* = 0.0395) in the sensitivity test, the HPTA value was not significantly associated with global cognition in the total sample and the sensitivity test for MCI and pre-MCI groups. The mediation effect on the association between the SNR and cognitive performance was not observed in this study. A possible explanation is that hearing impairment in a high frequency occurs early and is sensitive to cardiometabolic risk factors. Further investigation is needed in following issues using a large sample and longitudinal cohort. Whether or not CMM participated the mediation effects of the relationships peripheral or CAPD and cognitive impairment. Whether or not the mechanisms of CMM, including vascular impairment, systemic chronic inflammation, insulin resistance, and cellular energy metabolism dysfunction also resulted in peripheral hearing loss, CAPD, and brain. Nonetheless, to our knowledge, this is one of the first studies to examine the impact of CMM on global and domain-specific cognition by modifying peripheral and central hearing loss risk.

The above results suggest the importance of multidisciplinary cooperation in clinical practice to prevent and reduce hearing and cardiometabolic risk factors in the early stage. Active preventive efforts to target and reduce hearing loss and CMM should be recognized as reducing the risk of cognitive impairment. Optimizing central auditory processing and cardiometabolic health, especially in individuals with preclinical cognitive impairment in the middle-life period, can promote cognitive performance and lead to improved benefits later in life. Recently, a multicenter, randomized control trial verified that hearing aid amplification could reduce cognitive changes over 3 years in populations of older adults at increased risk for cognitive decline (Lin et al., 2023). The trial results support our findings that a high-frequency PTA decline was independently associated with processing speed/attention and executive function. However, auditory rehabilitation could benefit from enhancing not only audibility through the provision of hearing aids but also auditory temporal processing and other auditory cognitive functions, such as memory and language functions (Humes, 2021; Johnson et al., 2021). Auditory perceptual training involving word-based training (Humes et al., 2014), phoneme discrimination in quiet and noise, and working memory skills (Ferguson and Henshaw, 2015) have shown training-related benefits. An active integrated lifestyle (e.g., physical exercise and leisure activities, social interaction, high sleep quality, and no smoking and alcohol uptake) has been shown to mitigate the dose–response relationship between CMD status and the risk of early cognitive decline (Li H. et al., 2024). Reducing vascular and cardiometabolic risk factors, including low physical activity, can significantly decrease all-cause dementia risk, especially among ApoE*4 carriers (Shaaban et al., 2019). Therefore, integrated health care management and personalized intervention based on peripheral and central auditory dysfunction, CMDs, and cognitive status using sound amplification by hearing aids, a global cognitive training approach, and CMD management will be beneficial.

The major strength of this study is that global and domain-specific cognitive performance was assessed on the basis of a comprehensive neuropsychological test battery. Another strength is the use of LPTA, HPTA, and SNR loss in the better ear based on LPTA, as an entity to define the association between peripheral hearing loss or CAPD and global and domain-specific cognitive performance, which is modified by CMM. Additionally, *p*-value correction and regression models in this study adjust for LPTA, HPTA, and SNR; auditory function and CMDs; and frailty, BMI, social dysfunction, lifestyle, frailty status, and tinnitus THI, GDS, and NPI scores. The peripheral and central hearing measure methods in our study were introduced temporal gaps between the target stimuli, which is highly effective in ensuring the patient’s response reflects the external sound perception rather than confusion with the internal tinnitus percept. This method also was confirmed to be reliable and could exclude the impact of chronic tinnitus on the perception of external sounds, including basic auditory tasks and speech perception in noise (Zeng et al., 2020). Nevertheless, a major limitation is to perform mediation in a cross-sectional sample, which not only limit to establish temporal and causal relationships of CMM, peripheral or central hearing loss and cognitive impairment, but also limit the robustness of CMM mediating the relationships between peripheral or central hearing loss and cognitive impairment. The temporal impact of collecting variables on mediation analysis could not be excluded in our cross-sectional cohort. For example, the variables of participants were collected at an average age of 72 or above. However, some participants might experience CMDs in middle age. The temporal inconsistence of collecting data of CMDs and the presence of CMDs will affect the accuracy of mediation analysis. The findings from these mediation analyses are preliminary, exploratory, and hypothesis-generating in nature, and that the causal direction cannot be established within a cross-sectional design. Therefore, a longitudinal cohort study is need to verify the conclusions in the present study. The limited sample size also largely influences the robustness of results in this study. Another limitation is that our all-Han nationality study cohort reflects only the urban older adult population from a single specific location. The generalizability of the findings may be limited, and needs to be verified in other types of populations with a large sample. In addition, although most covariates were included in the cross-sectional study, some residual confounders (e.g., more than 20% data missing for nutrition and static balance status were excluded in the study) or potential mediators [e.g., genetic predisposition, socioeconomic status, other chronic morbidities (hypothyroidism), etc.] that were not available in this study could not be fully accounted for. Moreover, a methodological limit is that background noise in the CAP test is not speech noise that replicates real-life listening situations. The speech signals were presented monaurally, and it was impossible to assess binaural interactions. Finally, the results of this study revealed only a dose–response relationship between CMDs and LPTA, HPTA, or SNR values and did not reflect the effects of specific CMDs or the combination of CMDs on cognitive performance. The results also did not reflect the concrete effects of peripheral hearing loss or CAPD on cognitive performance because the LPTA, HPTA, and SNR values were analyzed as continuous variables and stratified into tertiles by performance strata.

In conclusion, our data from a cross-sectional study of community-dwelling older people support and complement those of previous studies by indicating independent and joint effects of CMM and LPTA, HPTA, or CAPD on global and domain-specific cognitive performance. These findings suggest important roles of the early prevention and management of CMDs in delaying global and domain-specific cognitive impairment in older people, in addition to the important role of auditory-related interventions. Future studies could investigate the association between baseline CMM and peripheral, central hearing decline, and cognitive decline over time, and the joint effect on cognition of baseline CMM and hearing loss, as well as the mediating effect of baseline CMM on the association between peripheral or central hearing loss and cognition.

## Data Availability

The original contributions presented in the study are included in the article/Supplementary material, further inquiries can be directed to the corresponding authors.

## Supplementary material

SUPPLEMENTARY FIGURE 1 Relationship between HPTA, CMM, and global or domain-specific cognitive performance in the sensitivity test.
