# Supplementary material for: Peripheral and central auditory dysfunction, cardiometabolic multimorbidity, and cognitive performance in community-dwelling older adults: a cross-sectional study
Source: Front Neurosci. 2026 Jan 16;19:1646313. doi: 10.3389/fnins.2025.1646313 (PMC12856757; doi:10.3389/fnins.2025.1646313)
Supplement: Supplementary file 2 [file Table_1.docx]

Supplementary Table 1. Characteristics of the study population by cognitive status (n = 509)

| Characteristic | Cognitively normal control (n = 193) | Pre-MCI (n =144) | MCI  (n = 171) | Adjusted P  （PreMCI vs Control） | Adjusted P  (MCI vs Control) | Adjusted P  (PreMCI vs MCI) |
| --- | --- | --- | --- | --- | --- | --- |
| Age (yrs) | 72.00(68.00-77.00) | 73.00(69.00-78.00) | 73.00(69.00-78.75) | 1.000 | 1.000 | 1.000 |
| Male sex, % | 98 (50.78) | 53 (36.81) | 75 (43.86) | 1.000 | 1.000 | 1.000 |
| Education |  |  |  | 1.000 | 9.680e-04 | 1.000 |
| Illiterate or primary education, % | 2 (1.04) | 7 (4.86) | 18 (10.53) |  |  |  |
| Middle school education, % | 111 (57.51) | 87 (60.42) | 111 (64.91) |  |  |  |
| College education or more, % | 77 (39.90) | 50 (34.72) | 41 (23.98) |  |  |  |
| Cardiovascular disease, % | 95 (49.22) | 99 (68.75) | 116 (67.84) | 0.0376 | 0.0503 | 1.000 |
| Diabetes,% | 28 (14.51) | 31 (21.53) | 44 (25.73) | 1.000 | 1.000 | 1.000 |
| Stroke, % | 13 (6.74) | 9 (6.25) | 24 (14.04) | 4.717e-21 | 1.000 | 1.000 |
| Non-skin malignancy, % | 28 (14.51) | 8 (5.56) | 17 (9.94) | 1.000 | 1.000 | 1.000 |
| The number of cardometabolic multimorbidity | 2.00(1.00-3.00) | 2.00(1.00-3.00) | 2.00(1.00-3.00) | 1.000 | 1.000 | 1.000 |
| BMI | 24.01(21.67-25.80) | 24.02(21.49-25.87) | 24.22 (22.00-26.10) | 1.000 | 1,000 | 1.000 |
| Smoking, % |  |  |  | 1.000 | 1.000 | 1.000 |
| No smoking | 140 (72.54) | 121 (84.03) | 136 79.53）） |  |  |  |
| Former | 22 (11.40) | 8 (5.56) | 17 (9.94) |  |  |  |
| Current | 15 (7.77) | 9 (6.25) | 13 (7.60) |  |  |  |
| Alcohol use, % |  |  |  | 1.000 | 1.000 | 1.000 |
| No alcohol | 150 (77.72) | 129 (89.58) | 143 (83.63) |  |  |  |
| Former | 15 (7.77) | 5 (3.47) | 9 (5.26) |  |  |  |
| Current | 13 (6.74) | 4 (2.78) | 14 (8.19) |  |  |  |
| Living alone, % | 14.00(7.25) | 14.00(9.72) | 15.00(8.77） | 1.000 | 1.000 | 1.000 |
| Social dysfunction score | 27.00(23.00-32.00） | 28.00(23.00-34.00） | 28.00(24.00-36.00） | 1.000 | 1.000 | 1.000 |
| GDS15 score | 3.00(1.00-5.00) | 3.00(1.50-5.00) | 3.00 (2.00-5.00) | 1.000 | 1.000 | 1.000 |
| MMSE score | 28.00(27.00-29.00) | 27.00(26.00-28.00) | 26.00(25.00-28.00) | 1.141e-03 | 4.624e-11 | 0.0646 |
| NPI score | 0.00 (0.00-2.00) | 1.00(0.00-3.00） | 1.00(0.00-3.00） | 1.000 | 1.000 | 1.000 |
| THI score | 0.00(0.00-24.00) | 2.00(0.00-26.00) | 0.00 (0.00-22.00) | 1.000 | 1.000 | 1.000 |
| Frailty score | 1.00(0.00-1.00） | 1.00(0.00-2.00) | 1.00 (0.00-2.00) | 1.000 | 0.174 | 1.000 |
| Low_Frq PTA | 25.00(18.30-35.00) | 28.30(21.70-38.00) | 31.70(25.00-45.00) | 1.000 | 8.307e-04 | 0.295 |
| High_Frq PTA | 47.50 (32.50-60.00) | 50.00(36.70-62.50) | 55.00 (40.00-65.00) | 1.000 | 0.0160 | 1.000 |
| SNR | -4.40 (-5.20- -2.80) | -3.60 (-5.20- -1.20) | -2.80 (-4.40- 0.40) | 1.000 | 2.781e-04 | 1.000 |
| Attention/executive domain decline (TMT A & B), % | 10 (5.18) | 45 (31.25) | 107 (62.57) | 3.296e-08 | 3.94e-29 | 1.000 |
| Memory domain decline (Delayed recall & recognition), % | 45(23.32) | 91 (63.19) | 144 (84.21) | 3.036e-11 | 1.106e-28 | 1.000 |
| Language domain decline (BNT & animal fluency test), % | 19 (9.84) | 67 (46.53) | 118 (69.01) | 3.036e-11 | 1.106e-28 | 1.000 |

Medians and interquartile ranges [Q25–Q75] for continuous variables, and frequencies or percentages for categorical variables. Low_Frq, low-frequency; high_Frq, High-frequency; BMI, body mass index; MMSE, the Mini-Mental Status Exam; MCI, mild cognitive impairment; GDS, the Geriatric Depression Scale; NPI, self-report Neuropsychiatric Inventory Questionnaire; PTA, pure tone average; SNR, signal-to-noise ratio; BNT, Boston naming test; THI, Tinnitus Handicap Inventory. To account for multiple testing, we adjusted the significance level using the Bonferroni method.
