# Supplementary material for: Peripheral and central auditory dysfunction, cardiometabolic multimorbidity, and cognitive performance in community-dwelling older adults: a cross-sectional study
Source: Front Neurosci. 2026 Jan 16;19:1646313. doi: 10.3389/fnins.2025.1646313 (PMC12856757; doi:10.3389/fnins.2025.1646313)
Supplement: Supplementary file 3 [file Table_2.docx]

Supplementary Table 2-1. Characteristics of the study population by cognitive status (n = 392)

| Characteristic | Cognitively normal control (n = 158) | Pre-MCI (n =118) | MCI  (n = 116) | P  （PreMCI vs Control） | P  (MCI vs Control) | P  (PreMCI vs MCI) |
| --- | --- | --- | --- | --- | --- | --- |
| Age (yrs), mean (SD) | 71.00 (66.25-76.00) | 73.00 (68.25-77.00) | 73.00 (68.00-77.50) | 1.000 | 1.000 | 1.000 |
| Male sex, % | 82 (51.90) | 42 (35.59) | 51 (43.97) | 0.850 | 1.000 | 1.000 |
| Education |  |  |  | 1.000 | 5.91e-03 | 1.000 |
| Illiterate or primary education, % | 2 (1.27) | 6 (5.08) | 13 (11.21) |  |  |  |
| Middle school education, % | 90 (56.96) | 73 (61.86) | 76 (65.52) |  |  |  |
| College education or more, % | 61 (38.61) | 39 (33.05) | 26 (22.41) |  |  |  |
| Cardiovascular disease, % | 78 (49.37) | 81 (68.64) | 79 (68.10) | 0.152 | 0.294 | 1.000 |
| Diabetes,% | 21 (13.29) | 24 (20.34) | 30 (25.86) | 1.000 | 1.000 | 1.000 |
| Stroke, % | 11 (6.96) | 8 (6.78) | 19 (16.38) | 7.455e-17 | 1.000 | 1.000 |
| Non-skin malignancy, % | 24 (15.19) | 7 (5.93) | 10 (8.62) | 1.000 | 1.000 | 1.000 |
| The number of cardometabolic multimorbidity | 2.00(1.00-3.00) | 2.00(1.00-3.00) | 2.00(1.00-3.00) | 1.000 | 1.000 | 1.000 |
| BMI | 24.00(21.64-25.80) | 24.17(22.10-25.95) | 24.22(22.05-26.10) | 1.000 | 1.000 | 1.000 |
| Smoking, % |  |  |  | 1.000 | 1.000 | 1.000 |
| No smoking | 115 (72.78) | 99 (83.90) | 91 (78.45) |  |  |  |
| Former | 18 (11.39) | 6 (5.08) | 11 (9.48) |  |  |  |
| Current | 13 (8.23) | 9 (7.63) | 11 (9.48) |  |  |  |
| Alcohol use, % |  |  |  | 1.000 | 1.000 | 1.000 |
| No alcohol | 124 (78.48) | 107 (90.68) | 92 (79.31) |  |  |  |
| Former | 13 (8.23) | 4 (3.39) | 8 (6.90) |  |  |  |
| Current | 10 (6.33) | 3 (2.54) | 13 (11.21) |  |  |  |
| Living alone, % | 14 (8.86) | 13 (11.02) | 11 (9.48) | 1.000 | 1.000 | 1.000 |
| Social dysfunction score | 27.00(23.00-32.00) | 28.00(23.75-34.00) | 27.00(23.00-33.25) | 1.000 | 1.000 | 1.000 |
| GDS15 score | 3.00 (1.00-5.00) | 3.00(1.00-5.00) | 3.00(2.00-5.00) | 1.000 | 1.000 | 1.000 |
| MMSE score | 28.00(27.00-29.00) | 27.00 (26.00-28.00) | 27.00 (25.00-28.00) | 2.316e-03 | 7.932e-09 | 0.440 |
| NPI score | 0.00 (0.00-2.00) | 1.00(0.00-3.00) | 1.00 (0.00-2.25) | 1.000 | 1.000 | 1.000 |
| THI score | 0.00(0.00-22.00) | 0.00(0.00-22.50) | 0.00 (0.00-10.50) | 1.000 | 1.000 | 1.000 |
| Frailty score | 1.00 (0.00-1.00) | 1.00(0.00-2.00) | 1.00(0.00-2.00) | 1.000 | 1.000 | 1.000 |
| Low_Frq PTA | 23.30(17.00-30.00) | 26.70(20.00-31.70) | 28.00(21.27-33.00) | 1.000 | 0.143 | 1.000 |
| High_Frq PTA | 42.50(27.50-55.00) | 45.00(33.72-56.48) | 50.85(35.00-60.00) | 1.000 | 0.220 | 1.000 |
| SNR | -4.40(-5.20- -3.60) | -3.60(-5.20- -2.20) | -3.60 (-5.20- -2.00) | 1.000 | 0.365 | 1.000 |
| Attention/executive domain decline (TMT A & B), % | 8 (5.06) | 34 (28.81) | 72 (62.07) | 1.177e-05 | 3.971e-22 | 1.000 |
| Memory domain decline (Delayed recall & recognition), % | 37 (23.42) | 74 (62.71) | 102 (87.93) | 8.660e-09 | 1.496e-23 | 1.000 |
| language domain decline (BNT & animal fluency test), % | 15 (9.49) | 54 (45.76) | 83 (71.55) | 1.297e-09 | 1.089e-23 | 1.000 |

Medians and interquartile ranges [Q25–Q75] for continuous variables, and frequencies or percentages for categorical variables. Low_Frq, low-frequency; high_Frq, High-frequency; BMI, body mass index; MMSE, the Mini-Mental Status Exam; MCI, mild cognitive impairment; GDS, the Geriatric Depression Scale; NPI, self-report Neuropsychiatric Inventory Questionnaire; PTA, pure tone average; SNR, signal-to-noise ratio; BNT, Boston naming test; THI, Tinnitus Handicap Inventory. To account for multiple testing, we adjusted the significance level using the Bonferroni method.
