# Supplementary material for: Peripheral and central auditory dysfunction, cardiometabolic multimorbidity, and cognitive performance in community-dwelling older adults: a cross-sectional study
Source: Front Neurosci. 2026 Jan 16;19:1646313. doi: 10.3389/fnins.2025.1646313 (PMC12856757; doi:10.3389/fnins.2025.1646313)
Supplement: Supplementary file 5 [file Table_4.docx]

Supplementary Table 3. Association of overall cognitive performance with hearing loss of low_frq, high_frq, and auditory processing in the better ear, or CMM

|  | Variable | Total sample test Model 1 | | | Sensitivity test Model 1 | | |
| --- | --- | --- | --- | --- | --- | --- | --- |
|  |  | β（95%CI） | P value | Adjusted P value | β（95%CI） | P value | Adjusted  P value |
| Pre-MCI  Vs  Cognitively intact | Low_Frq PTA | 0.002 (-0.002, 0.006) | 0.369 | 0.381 | 0.006 (-0.002, 0.013) | 0.136 | 0.217 |
|  | High_Frq PTA | 0.003 (-0.001, 0.006) | 0.103 | 0.143 | 0.004 (0.000, 0.009) | 0.0324 | 0.065 |
|  | SNR | 0.012 (-0.004, 0.028) | 0.141 | 0.169 | 0.017 (-0.007, 0.040) | 0.166 | 0.217 |
|  | CMM (Continuous) | 0.121 (0.047, 0.196) | 0.0016 | 0.0038 | 0.132 (0.048, 0.215) | 2.223e-03 | 0.0091 |
|  | CMM = 0 | Ref |  |  | Ref |  |  |
|  | CMM = 1 | 0.093 (-0.026, 0.211) | 0.1274 | 0.164 | 0.094 (-0.039, 0.227) | 0.169 | 0.217 |
|  | CMM >=2 | 0.249 (0.096, 0.403) | 0.0017 | 0.0038 | 0.263 (0.090, 0.437) | 3.501e-03 | 0.0091 |
| MCI  Vs  Cognitively intact | Low_Frq PTA | 0.007 (0.004, 0.011) | 0.00012 | 0.00072 | 0.011 (0.004, 0.019) | 3.537e-03 | 0.0091 |
|  | High_Frq PTA | 0.006 (0.003, 0.009) | 0.00025 | 0.0011 | 0.007 (0.003, 0.011) | 5.290e-04 | 0.0032 |
|  | SNR | 0.025 (0.012, 0.039) | 0.00034 | 0.0012 | 0.031 (0.011, 0.051) | 3.116e-03 | 0.0091 |
|  | CMM (Continuous) | 0.160 (0.086, 0.234) | 2.78E-05 | 0.00025 | 0.176 (0.094, 0.257) | 3.180E-05 | 0.00029 |
|  | CMM = 0 | Ref |  |  | Ref |  |  |
|  | CMM = 1 | 0.195 (0.078, 0.312) | 1.26e-03 | 0.0038 | 0.153 (0.023, 0.283) | 0.0216 | 0.049 |
|  | CMM >=2 | 0.332 (0.187, 0.478) | 1.31E-05 | 0.00024 | 0.355 (0.196, 0.514) | 2.54E-05 | 0.00029 |
| MCI  Vs  Pre-MCI | Low_Frq PTA | 0.006 (0.002, 0.009) | 0.0065 | 0.013 | 0.003 (-0.005, 0.012) | 0.459 | 0.459 |
|  | High_Frq PTA | 0.003 (0.000, 0.007) | 0.0596 | 0.098 | 0.002 (-0.002, 0.007) | 0.299 | 0.317 |
|  | SNR | 0.013 (-0.001, 0.028) | 0.0708 | 0.106 | 0.013 (-0.008, 0.034) | 0.229 | 0.258 |
|  | CMM (Continuous) | 0.037 (-0.043, 0.118) | 0.362 | 0.381 | 0.061 (-0.029, 0.151) | 0.185 | 0.222 |
|  | CMM = 0 | Ref |  |  | Ref |  |  |
|  | CMM = 1 | 0.135 (-0.002, 0.272) | 0.055 | 0.098 | 0.119 (-0.037, 0.274) | 0.136 | 0.217 |
|  | CMM >=2 | 0.073 (-0.089, 0.235) | 0.381 | 0.381 | 0.127 (-0.053, 0.306) | 0.169 | 0.217 |

CMM, cardiometabolic multimorbidity; CI, Confidence Interval; Low_Frq, low-frequency; high_Frq, High-frequency; MCI, mild cognitive impairment; PTA, pure tone average; SNR, signal-to-noise ratio.
