# Supplementary material for: Peripheral and central auditory dysfunction, cardiometabolic multimorbidity, and cognitive performance in community-dwelling older adults: a cross-sectional study
Source: Front Neurosci. 2026 Jan 16;19:1646313. doi: 10.3389/fnins.2025.1646313 (PMC12856757; doi:10.3389/fnins.2025.1646313)
Supplement: Supplementary file 6 [file Table_5.docx]

Supplementary Table 4. Association of domain-specific cognitive performance with hearing loss of low- and high- frequency PTA, and SNR in the better ear, or CMM

| Domain | Variables | Total sample Model 1 | |  | Sensitivity test Model 1 | |  |
| --- | --- | --- | --- | --- | --- | --- | --- |
|  |  | β （95%CI） | P value | Adjusted p value | β（95%CI） | P value | Adjusted p value |
| TMT A | Low_Frq PTA | 0.003 (0.000, 0.006) | 0.0215 | 0.059 | 0.005 (0.000, 0.010) | 0.0479 | 0.112 |
|  | High_Frq PTA | 0.003 (0.001, 0.005) | 0.0081 | 0.035 | 0.003 (0.001, 0.006) | 0.0153 | 0.054 |
|  | SNR | 0.008 (-0.002, 0.018) | 0.108 | 0.180 | 0.005 (-0.010, 0.019) | 0.523 | 0.704 |
|  | CMM (Continuous) | 0.061 (0.009, 0.114) | 0.0212 | 0.059 | 0.059 (0.004, 0.115) | 0.037 | 0.101 |
|  | CMM =0 | Ref |  |  | Ref |  |  |
|  | CMM = 1 | 0.028 (-0.053, 0.110) | 0.492 | 0.574 | -0.001 (-0.085, 0.084) | 0.986 | 0.994 |
|  | CMM >=2 | 0.137 (0.032, 0.242) | 0.011 | 0.040 | 0.123 (0.007, 0.239) | 0.0398 | 0.101 |
| TMT B | Low_Frq PTA | 0.001 (-0.001, 0.004) | 0. 265 | 0.371 | 0.003 (-0.002, 0.008) | 0. 280 | 0.458 |
|  | High_Frq PTA | 0.002 (0.000, 0.005) | 0.0266 | 0.067 | 0.004 (0.001, 0.006) | 6.779e-03 | 0.032 |
|  | SNR | 0.001 (-0.009, 0.010) | 0.908 | 0.935 | 0.001 (-0.013, 0.015) | 0.902 | 0.957 |
|  | CMM (Continuous ) | 0.096 (0.045, 0.146) | 2.37e-04 | 0.0026 | 0.117 (0.061, 0.172) | 4.610e-05 | 0.00054 |
|  | CMM =0 | Ref |  |  | Ref |  |  |
|  | CMM = 1 | 0.147 (0.068, 0.226) | 2.930e-04 | 0.0026 | 0.181(0.096, 0.266) | 4.040e-05 | 0.00054 |
|  | CMM >=2 | 0.181 (0.091, 0.272) | 1.06E-04 | 0.0019 | 0.217 (0.119, 0.314) | 2.280e-05 | 0.00054 |
| Delayed recall  Of HVLT-R | Low_Frq PTA | 0.006 (0.003, 0.008) | 4.91e-05 | 0.0017 | 0.007 (0.002, 0.012) | 7.753e-03 | 0.0317 |
|  | High_Frq PTA | 0.003 (0.001, 0.005) | 4.56e-03 | 0.0046 | 0.002 (0.000, 0.005) | 0.078 | 0.161 |
|  | SNR | 0.018 (0.008, 0.028) | 3.74e-04 | 0.0026 | 0.020 (0.005, 0.034) | 8.150e-03 | 0.0317 |
|  | CMM (Continuous) | 0.030 (-0.023, 0.082) | 0.270 | 0.374 | 0.023 (-0.034, 0.081) | 0.430 | 0.627 |
|  | CMM = 0 | Ref |  |  | Ref |  |  |
|  | CMM = 1 | 0.045 (-0.04, 0.130) | 0.299 | 0.374 | 0.013 (-0.080, 0.106) | 0.778 | 0.851 |
|  | CMM >=2 | 0.066 (-0.039, 0.171) | 0.221 | 0.336 | 0.057 (-0.060, 0.174) | 0.344 | 0.547 |
| Recognition of HVLT-R | Low_Frq PTA | 0.000 (-0.003, 0.003) | 0.889 | 0.935 | 0.002 (-0.004, 0.009) | 0.523 | 0.704 |
|  | High_Frq PTA | 0.001 (-0.001, 0.004) | 0.293 | 0.374 | 0.002 (-0.001, 0.006) | 0.187 | 0.325 |
|  | SNR | 0.007 (-0.005, 0.019) | 0.271 | 0.374 | 0.016 (-0.002, 0.034) | 0.088 | 0.171 |
|  | CMM (Continuous) | 0.054 (-0.009, 0.117) | 0.095 | 0.175 | 0.047 (-0.024, 0.118) | 0.195 | 0.325 |
|  | CMM = 0 | Ref |  |  | Ref |  |  |
|  | CMM = 1 | 0.056 (-0.046, 0.158) | 0.279 | 0.374 | 0.000 (-0.115, 0.115) | 0.994 | 0.994 |
|  | CMM >=2 | 0.107 (-0.022, 0.235) | 0.105 | 0.180 | 0.102 (-0.044, 0.248) | 0.172 | 0.317 |
| BNT | Low_Frq PTA | 0.003 (0.000, 0.006) | 0.0527 | 0.115 | 0.003 (-0.003, 0.008) | 0.372 | 0.566 |
|  | High_Frq PTA | 0.001 (-0.001, 0.003) | 0.320 | 0.386 | 0.001 (-0.002, 0.004) | 0.638 | 0.811 |
|  | SNR | 0.011 (0.001, 0.021) | 0.035 | 0.082 | 0.003 (-0.012, 0.018) | 0.704 | 0.838 |
|  | CMM (Continuous) | 0.053 (-0.002, 0.107) | 0.0590 | 0.115 | 0.063 (0.003, 0.124) | 0.0402 | 0.101 |
|  | CMM = 0 | Ref |  |  | Ref |  |  |
|  | CMM = 1 | 0.020 (-0.066, 0.106) | 0.643 | 0.703 | 0.022 (-0.073, 0.118) | 0.649 | 0.811 |
|  | CMM >=2 | 0.108 (-0.004, 0.220) | 0.059 | 0.115 | 0.129 (0.007, 0.252) | 0.0397 | 0.101 |
| Animal fluency | Low_Frq PTA | 0.002 (-0.001, 0.005) | 0.215 | 0.336 | 0.001 (-0.005, 0.007) | 0.742 | 0.838 |
|  | High_Frq PTA | 0.001 (-0.002, 0.003) | 0.613 | 0.692 | 0.001 (-0.003, 0.004) | 0.730 | 0.838 |
|  | SNR | 0.000 (-0.011, 0.011) | 0.996 | 0.996 | -0.015 (-0.031, 0.001) | 0.0616 | 0.135 |
|  | CMM (Continuous) | 0.073 (0.017, 0.130) | 0.0115 | 0.040 | 0.099 (0.037, 0.162) | 0.002 | 0.014 |
|  | CMM = 0 | Ref |  |  | Ref |  |  |
|  | CMM = 1 | 0.152 (0.063, 0.242) | 9.620e-04 | 0.0056 | 0.170 (0.070, 0.269) | 9.260e-04 | 0.0081 |
|  | CMM >=2 | 0.129 (0.019, 0.239) | 0.0219 | 0.059 | 0.178 (0.056, 0.300) | 4.691e-03 | 0.027 |

CMM, cardiometabolic multimorbidity; CI, Confidence Interval; Low_Frq, low-frequency; high_Frq, High-frequency; PTA, pure tone average; SNR, signal-to-noise ratio; HVLT-R, the Hopkins Verbal Learning Test-Revised; BNT, Boston Naming Test; TMT Part A and B, Trail Making Test A and B.
