# Supplementary material for: Peripheral and central auditory dysfunction, cardiometabolic multimorbidity, and cognitive performance in community-dwelling older adults: a cross-sectional study
Source: Front Neurosci. 2026 Jan 16;19:1646313. doi: 10.3389/fnins.2025.1646313 (PMC12856757; doi:10.3389/fnins.2025.1646313)
Supplement: Supplementary file 7 [file Table_6.docx]

Supplementary Table 5. The joint effects of LPTA, HPTA, or SNR and CMD stratification on global cognitive performance in Model 1

| Groups |  | Total sample Model 1 | |  | Sensitivity test Model 1 | |  |
| --- | --- | --- | --- | --- | --- | --- | --- |
|  |  | β （95%CI） | P value | Adjusted P value | β（95%CI） | P value | Adjusted P value |
| Pre-MCI vs  Cognitively normal control | **Low_Frq** |  |  |  |  |  |  |
|  | Tertile1, CMD=0 | Ref |  |  | Ref |  |  |
|  | Tertile1, CMD=1 | 0.003 (0.000, 0.006) | 0.339 | 0.469 | 0.086 (-0.123, 0.294) | 0.423 | 0.565 |
|  | Tertile1, CMD≥2 | 0.097 (-0.101, 0.295) | 0.0052 | 0.025 | 0.335 (0.016, 0.655) | 0.045 | 0.147 |
|  | Tertile2, CMD=0 | 0.045 (-0.172, 0.262) | 0.685 | 0.780 | 0.063 (-0.162, 0.288) | 0.584 | 0.690 |
|  | Tertile2, CMD=1 | 0.194 (-0.013, 0.402) | 0.0696 | 0.150 | 0.264 (0.034, 0.494) | 0.027 | 0.114 |
|  | Tertile2, CMD≥2 | 0.333 (0.075, 0.590) | 0.014 | 0.0438 | 0.373 (0.116, 0.630) | 6.21e-03 | 0.0402 |
|  | Tertile3, CMD=0 | -0.023 (-0.259, 0.214) | 0.852 | 0.876 | 0.112 (-0.158, 0.383) | 0.419 | 0.565 |
|  | Tertile3, CMD=1 | 0.038 (-0.175, 0.251) | 0.728 | 0.795 | 0.101 (-0.123, 0.326) | 0.379 | 0.557 |
|  | Tertile3, CMD≥2 | 0.202 (-0.082, 0.484) | 0.889 | 0.902 | 0.347 (0.030, 0.664) | 0.037 | 0.127 |
|  | **High_Frq** |  |  |  |  |  |  |
|  | Tertile1, CMD= 0 | Ref |  |  | Ref |  |  |
|  | Tertile1, CMD=1 | 0.191 (-0.007, 0.388) | 0.062 | 0.144 | 0.134 (-0.081, 0.349) | 0.225 | 0.421 |
|  | Tertile1, CMD≥2 | 0.292 (0.023, 0.562) | 0.038 | 0.094 | 0.252 (-0.083, 0.588) | 0.147 | 0.338 |
|  | Tertile2, CMD=0 | 0.113 (-0.086, 0.311) | 0.268 | 0.398 | 0.084 (-0.125, 0.294) | 0.433 | 0.567 |
|  | Tertile2, CMD=1 | 0.036 (-0.176, 0.247) | 0.742 | 0.797 | 0.108 (-0.111, 0.328) | 0.337 | 0.506 |
|  | Tertile2, CMD≥2 | 0.499 (0.231, 0.768) | 5.46e-04 | 0.0044 | 0.484 (0.238, 0.730) | 2.94e-04 | 0.0084 |
|  | Tertile3, CMD=0 | 0.080 (-0.178, 0.338) | 0.544 | 0.664 | 0.184 (-0.095, 0.463) | 0.201 | 0.413 |
|  | Tertile3, CMD=1 | 0.200 (-0.018, 0.418) | 0.075 | 0.154 | 0.177 (-0.065, 0.418) | 0.155 | 0.338 |
|  | Tertile3, CMD≥2 | 0.150 (-0.124, 0.432) | 0.287 | 0.408 | 0.137 (-0.181, 0.456) | 0.402 | 0.565 |
|  | **SNR** |  |  |  |  |  |  |
|  | Tertile1, CMD=0 | Ref |  |  | Ref |  |  |
|  | Tertile1, CMD=1 | 0.237 (0.037, 0.438) | 0.023 | 0.064 | 0.230 (0.022, 0.438) | 0.033 | 0.127 |
|  | Tertile1, CMD≥2 | 0.354 (0.104, 0.603) | 7.30e-03 | 0.0292 | 0.331 (0.067, 0.596) | 0.017 | 0.0816 |
|  | Tertile2, CMD=0 | 0.031 (-0.172, 0.234) | 0.764 | 0.809 | -0.052(-0.284, 0.180) | 0.664 | 0.742 |
|  | Tertile2, CMD=1 | 0.038 (-0.149, 0.225) | 0.690 | 0.780 | 0.018 (-0.192, 0.229) | 0.865 | 0.865 |
|  | Tertile2, CMD≥2 | 0.295 (0.051, 0.538) | 0.021 | 0.060 | 0.177 (-0.107, 0.460) | 0.228 | 0.421 |
|  | Tertile3, CMD=0 | 0.129 (-0.099, 0.357) | 0.271 | 0.398 | 0.173 (-0.056, 0.402) | 0.143 | 0.338 |
|  | Tertile3, CMD=1 | 0.285 (0.062, 0.507) | 0.014 | 0.044 | 0.213 (-0.020, 0.446) | 0.078 | 0.230 |
|  | Tertile3, CMD≥2 | 0.219 (-0.080, 0.518) | 0.157 | 0.263 | 0.384 (0.063, 0.706) | 0.023 | 0.104 |
| MCI vs Cognitively normal control  Animal fluency | **Low_Frq** |  |  |  |  |  |  |
|  | Tertile1, CMD=0 | Ref |  |  | Ref |  |  |
|  | Tertile1, CMD=1 | 0.170 (-0.009, 0.349) | 0.066 | 0.149 | 0.216 (0.017, 0.415) | 0.037 | 0.127 |
|  | Tertile1, CMD≥2 | 0.302 (0.092, 0.512) | 0.0064 | 0.029 | 0.354 (0.110, 0.598) | 6.703e-03 | 0.040 |
|  | Tertile2, CMD=0 | 0.118 (-0.064, 0.301) | 0.208 | 0.333 | 0.174 (-0.025, 0.374) | 0.092 | 0.255 |
|  | Tertile2, CMD=1 | 0.391 (0.206, 0.577) | 7.39e-05 | 9.096e-04 | 0.371 (0.165, 0.578) | 6.98e-04 | 8.376e-03 |
|  | Tertile2, CMD≥2 | 0.585 (0.350, 0.820) | 7.87e-06 | 2.057e-04 | 0.455 (0.215, 0.695) | 5.09e-04 | 8.376e-03 |
|  | Tertile3, CMD=0 | 0.347 (0.127, 0.567) | 2.881e-03 | 0.017 | 0.143 (-0.107, 0.393) | 0.268 | 0.459 |
|  | Tertile3, CMD=1 | 0.452 (0.264, 0.643) | 8.57e-06 | 2.057e-04 | 0.328 (0.115, 0.542) | 3.627e-03 | 0.027 |
|  | Tertile3, CMD≥2 | 0.390 (0.142, 0.638) | 3.144e-03 | 0.017 | 0.436 (0.196, 0.675) | 8.46e-04 | 8.702e-03 |
|  | **High_Frq** |  |  |  |  |  |  |
|  | Tertile1, CMD= 0 | Ref |  |  | Ref |  |  |
|  | Tertile1, CMD=1 | 0.070 (-0.111, 0.250) | 0.450 | 0.559 | 0.16 (-0.045, 0.365) | 0.130 | 0.323 |
|  | Tertile1, CMD≥2 | 0.283 (0.049, 0.517) | 0.021 | 0.060 | 0.169 (-0.161, 0.499) | 0.322 | 0.501 |
|  | Tertile2, CMD=0 | 0.055 (-0.127, 0.237) | 0.559 | 0.671 | -0.116 (-0.313, 0.082) | 0.257 | 0.451 |
|  | Tertile2, CMD=1 | 0.250 (0.061, 0.438) | 0.011 | 0.038 | 0.170 (-0.048, 0.388) | 0.130 | 0.323 |
|  | Tertile2, CMD≥2 | 0.256 (0.012, 0.501) | 0.044 | 0.106 | 0.470 (0.216, 0.723) | 6.36e-04 | 8.376e-03 |
|  | Tertile3, CMD=0 | 0.171 (-0.099, 0.442) | 0.219 | 0.343 | 0.282 (0.024, 0.539) | 0.037 | 0.127 |
|  | Tertile3, CMD=1 | 0.403 (0.214, 0.592) | 5.98e-05 | 9.096e-04 | 0.380 (0.169, 0.591) | 6.73e-04 | 8.376e-03 |
|  | Tertile3, CMD≥2 | 0.398 (0.149, 0.646) | 2.64e-03 | 0.017 | 0.402 (0.142, 0.662) | 3.766e-03 | 0.027 |
|  | **SNR** |  |  |  |  |  |  |
|  | Tertile1, CMD=0 | Ref |  |  | Ref |  |  |
|  | Tertile1, CMD=1 | 0.227 (0.074, 0.379) | 4.137e-03 | 0.021 | 0.251 (0.052, 0.451) | 0.016 | 0.082 |
|  | Tertile1, CMD≥2 | 0.372 (0.180, 0.564) | 2.81e-04 | 2.529e-03 | 0.213 (-0.051, 0.477) | 0.121 | 0.323 |
|  | Tertile2, CMD=0 | 0.004 (-0.196, 0.203) | 0.972 | 0.972 | -0.036 (-0.257, 0.184) | 0.748 | 0.792 |
|  | Tertile2, CMD=1 | 0.270 (0.099, 0.440) | 2.432e-03 | 0.017 | 0.118 (-0.077, 0.312) | 0.239 | 0.430 |
|  | Tertile2, CMD≥2 | 0.336 (0.098, 0.573) | 7.097e-03 | 0.029 | 0.314 (0.068, 0.560) | 0.016 | 0.082 |
|  | Tertile3, CMD=0 | 0.384 (0.188, 0.579) | 2.27e-04 | 2.335e-03 | 0.153 (-0.063, 0.370) | 0.170 | 0.360 |
|  | Tertile3, CMD=1 | 0.506 (0.329, 0.682) | 1.47e-07 | 1.058e-05 | 0.346 (0.133, 0.559) | 2.088e-03 | 0.019 |
|  | Tertile3, CMD≥2 | 0.471 (0.250, 0.691) | 7.58e-05 | 9.096e-04 | 0.544 (0.304, 0.784) | 5.02e-05 | 3.614e-03 |
| Pre-MCI vs MCI | **Low_Frq** |  |  |  |  |  |  |
|  | Tertile1, CMD=0 | Ref |  |  | Ref |  |  |
|  | Tertile1, CMD=1 | 0.164 (-0.067, 0.395) | 0.168 | 0.275 | 0.138 (-0.115, 0.390) | 0.291 | 0.476 |
|  | Tertile1, CMD≥2 | 0.159 (-0.109, 0.427) | 0.251 | 0.385 | 0.219 (-0.074, 0.512) | 0.151 | 0.338 |
|  | Tertile2, CMD=0 | 0.037 (-0.249, 0.323) | 0.799 | 0.834 | 0.168 (-0.148, 0.485) | 0.304 | 0.486 |
|  | Tertile2, CMD=1 | 0.188 (-0.036, 0.411) | 0.104 | 0.192 | 0.246 (-0.010, 0.502) | 0.065 | 0.203 |
|  | Tertile2, CMD≥2 | 0.220 (-0.048, 0.488) | 0.113 | 0.203 | 0.165 (-0.131, 0.461) | 0.282 | 0.472 |
|  | Tertile3, CMD=0 | 0.360 (0.056, 0.663) | 0.025 | 0.067 | 0.195 (-0.188, 0.578) | 0.327 | 0.501 |
|  | Tertile3, CMD=1 | 0.286 (0.072, 0.500) | 0.0105 | 0.038 | 0.159 (-0.095, 0.412) | 0.225 | 0.421 |
|  | Tertile3, CMD≥2 | 0.269 (-0.025, 0.564) | 0.081 | 0.162 | 0.282 (-0.024, 0.589) | 0.080 | 0.230 |
|  | **High_Frq** |  |  |  |  |  |  |
|  | Tertile1, CMD= 0 | Ref |  |  | Ref |  |  |
|  | Tertile1, CMD=1 | 0.105 (-0.119, 0.329) | 0.362 | 0.483 | 0.044 (-0.220, 0.308) | 0.744 | 0.792 |
|  | Tertile1, CMD≥2 | 0.128 (-0.162, 0.419) | 0.391 | 0.512 | 0.089 (-0.234, 0.411) | 0.594 | 0.690 |
|  | Tertile2, CMD=0 | 0.217 (-0.076, 0.510) | 0.154 | 0.263 | -0.203 (-0.521, 0.116) | 0.221 | 0.421 |
|  | Tertile2, CMD=1 | 0.201 (-0.025, 0.428) | 0.085 | 0.165 | 0.060 (-0.215, 0.335) | 0.670 | 0.742 |
|  | Tertile2, CMD≥2 | 0.053 (-0.209, 0.315) | 0.693 | 0.780 | 0.028 (-0.271, 0.327) | 0.855 | 0.865 |
|  | Tertile3, CMD=0 | 0.058 (-0.267, 0.383) | 0.729 | 0.795 | 0.034 (-0.323, 0.390) | 0.854 | 0.865 |
|  | Tertile3, CMD=1 | 0.241 (0.021, 0.462) | 0.035 | 0.090 | 0.093 (-0.177, 0.363) | 0.502 | 0.620 |
|  | Tertile3, CMD≥2 | 0.396 (0.110, 0.681) | 0.0096 | 0.036 | 0.148 (-0.210, 0.506) | 0.424 | 0.565 |
|  | **SNR** |  |  |  |  |  |  |
|  | Tertile1, CMD=0 | Ref |  |  | Ref |  |  |
|  | Tertile1, CMD=1 | 1.159 (0.922, 1.457) | 0.148 | 0.260 | 0.051 (-0.211, 0.314) | 0.704 | 0.768 |
|  | Tertile1, CMD≥2 | 0.130 (-0.141, 0.401) | 0.352 | 0.478 | 0.118 (-0.227, 0.462) | 0.508 | 0.620 |
|  | Tertile2, CMD=0 | -0.086 (-0.399, 0.227) | 0.592 | 0.699 | 0.033 (-0.321, 0.386) | 0.858 | 0.865 |
|  | Tertile2, CMD=1 | 0.127 (-0.106, 0.361) | 0.289 | 0.408 | 0.076 (-0.198, 0.351) | 0.588 | 0.690 |
|  | Tertile2, CMD≥2 | 0.125 (-0.166, 0.416) | 0.405 | 0.521 | 0.117 (-0.146, 0.381) | 0.388 | 0.559 |
|  | Tertile3, CMD=0 | 0.260 (-0.016, 0.535) | 0.071 | 0.150 | 0.119 (-0.248, 0.487) | 0.487 | 0.618 |
|  | Tertile3, CMD=1 | 0.199 (-0.029, 0.428) | 0.092 | 0.174 | 0.205 (-0.078, 0.489) | 0.489 | 0.618 |
|  | Tertile3, CMD≥2 | 0.132 (-0.193, 0.457) | 0.430 | 0.543 | 0.282 (-0.083, 0.647) | 0.647 | 0.739 |
