# Supplementary material for: Peripheral and central auditory dysfunction, cardiometabolic multimorbidity, and cognitive performance in community-dwelling older adults: a cross-sectional study
Source: Front Neurosci. 2026 Jan 16;19:1646313. doi: 10.3389/fnins.2025.1646313 (PMC12856757; doi:10.3389/fnins.2025.1646313)
Supplement: Supplementary file 8 [file Table_7.docx]

Supplementary Table 6. The joint effects of LPTA, HPTA, or SNR and CMD stratification on domain-specific cognitive performance in Model 1

| Groups |  | Total sample Model 1 | |  | Sensitivity test Model 1 | |  |
| --- | --- | --- | --- | --- | --- | --- | --- |
|  |  | β （95%CI） | P value | Adjusted P value | β（95%CI） | P value | Adjusted P value |
| Processing by TMT A | **Low_Frq** |  |  |  |  |  |  |
|  | Tertile1, CMD=0 | Ref |  |  | Ref |  |  |
|  | Tertile1, CMD=1 | -0.004 (-0.117, 0.109) | 0.943 | 0.977 | 0.017(-0.1,0.135) | 0.773 | 0.848 |
|  | Tertile1, CMD≥2 | 0.088 (-0.074, 0.249) | 0.291 | 0.4604 | 0.114(-0.071,0.299) | 0.231 | 0.499 |
|  | Tertile2, CMD=0 | 0.011 (-0.120, 0.142) | 0.866 | 0.937 | 0.097(-0.043,0.237) | 0.177 | 0.447 |
|  | Tertile2, CMD=1 | 0.093 (-0.039, 0.225) | 0.170 | 0.324 | 0.146(0.001,0.29) | 5.014e-02 | 0.201 |
|  | Tertile2, CMD≥2 | 0.177 (0.008, 0.346) | 4.287e-02 | 0.137 | 0.091(-0.068,0.25) | 0.267 | 0.557 |
|  | Tertile3, CMD=0 | 0.067 (-0.095, 0.229) | 0.420 | 0.576 | 0.049(-0.126,0.224) | 0.588 | 0.713 |
|  | Tertile3, CMD=1 | 0.068 (-0.084, 0.220) | 0.381 | 0.543 | 0.003(-0.136,0.141) | 0.971 | 0.978 |
|  | Tertile3, CMD≥2 | 0.225 (0.015, 0.434) | 3.858e-02 | 0.136 | 0.321(0.111,0.532) | 3.855e-03 | 0.064 |
|  | **High_Frq** |  |  |  |  |  |  |
|  | Tertile1, CMD= 0 | Ref |  |  | Ref |  |  |
|  | Tertile1, CMD=1 | 0.001 (-0.115, 0.118) | 0.981 | 0.988 | 0.058(-0.065,0.181) | 0.355 | 0.609 |
|  | Tertile1, CMD≥2 | 0.074 (-0.088, 0.236) | 0.375 | 0.54 | 0.122(-0.059,0.302) | 0.192 | 0.463 |
|  | Tertile2, CMD=0 | -0.033 (-0.160, 0.093) | 0.610 | 0.726 | 0.049(-0.087,0.184) | 0.483 | 0.680 |
|  | Tertile2, CMD=1 | 0.055 (-0.079, 0.188) | 0.425 | 0.577 | 0.071(-0.067,0.209) | 0.317 | 0.585 |
|  | Tertile2, CMD≥2 | 0.107 (-0.063, 0.277) | 0.221 | 0.383 | 0.167(-0.002,0.336) | 5.627e-02 | 0.212 |
|  | Tertile3, CMD=0 | 0.102 (-0.007, 0.274) | 0.248 | 0.415 | 0.151(-0.027,0.329) | 0.100 | 0.300 |
|  | Tertile3, CMD=1 | 0.120 (-0.025, 0.265) | 0.106 | 0.257 | 0.118(-0.029,0.265) | 0.118 | 0.335 |
|  | Tertile3, CMD≥2 | 0.282 (0.081, 0.484) | 7.329e-03 | 0.0503 | 0.34(0.139,0.54) | 1.420e-03 | 0.034 |
|  | **SNR** |  |  |  |  |  |  |
|  | Tertile1, CMD=0 | Ref |  |  | Ref |  |  |
|  | Tertile1, CMD=1 | 0.035 (-0.075, 0.144) | 0.537 | 0.684 | 0.022(-0.121,0.165) | 0.763 | 0.848 |
|  | Tertile1, CMD≥2 | 0.129 (-0.021, 0.278) | 0.094 | 0.251 | 0.097(-0.104,0.297) | 0.347 | 0.604 |
|  | Tertile2, CMD=0 | -0.036 (-0.180, 0.107) | 0.621 | 0.733 | -0.137(-0.294,0.019) | 0.090 | 0.295 |
|  | Tertile2, CMD=1 | -0.020 (-0.138, 0.098) | 0.744 | 0.838 | -0.118(-0.255,0.019) | 0.095 | 0.297 |
|  | Tertile2, CMD≥2 | 0.130 (-0.038, 0.298) | 0.132 | 0.292 | -0.032(-0.216,0.152) | 0.734 | 0.831 |
|  | Tertile3, CMD=0 | 0.057 (-0.086, 0.201) | 0.435 | 0.585 | -0.027(-0.187,0.132) | 0.739 | 0.831 |
|  | Tertile3, CMD=1 | 0.103 (-0.050, 0.256) | 0.188 | 0.338 | -0.048(-0.202,0.105) | 0.541 | 0.689 |
|  | Tertile3, CMD≥2 | 0.181 (-0.002, 0.364) | 5.588e-02 | 0.171 | 0.232(0.014,0.449) | 4.068e-02 | 0.178 |
| Attention/executive function (by TMT B) | **Low_Frq** |  |  |  |  |  |  |
|  | Tertile1, CMD=0 | Ref |  |  | Ref |  |  |
|  | Tertile1, CMD=1 | 0.176 (0.059, 0.293) | 3.838e-03 | 0.035 | 0.166(0.041,0.291) | 1.087e-02 | 0.087 |
|  | Tertile1, CMD≥2 | 0.135 (0.007, 0.262) | 4.155e-02 | 0.1377 | 0.223(0.068,0.378) | 6.335e-03 | 0.065 |
|  | Tertile2, CMD=0 | 0.004 (-0.082, 0.090) | 0.932 | 0.973 | 0.044(-0.054,0.142) | 0.382 | 0.609 |
|  | Tertile2, CMD=1 | 0.209 (0.079, 0.340) | 2.078e-03 | 0.033 | 0.222(0.081,0.363) | 2.563e-03 | 0.053 |
|  | Tertile2, CMD≥2 | 0.291 (0.147, 0.435) | 1.453e-04 | 0.013 | 0.154(0.018,0.29) | 2.922e-02 | 0.149 |
|  | Tertile3, CMD=0 | 0.081 (-0.044, 0.206) | 0.207 | 0.364 | -0.036(-0.137,0.065) | 0.492 | 0.680 |
|  | Tertile3, CMD=1 | 0.126 (-0.011, 0.263) | 7.434e-02 | 0.223 | 0.192(0.049,0.335) | 1.004e-02 | 0.085 |
|  | Tertile3, CMD≥2 | 0.040 (-0.105, 0.184) | 0.590 | 0.72 | 0.303(0.129,0.477) | 1.084e-03 | 0.032 |
|  | **High_Frq** |  |  |  |  |  |  |
|  | Tertile1, CMD= 0 | Ref |  |  | Ref |  |  |
|  | Tertile1, CMD=1 | 0.082 (-0.022, 0.186) | 0.127 | 0.292 | 0.127(0.014,0.239) | 2.956e-02 | 0.149 |
|  | Tertile1, CMD≥2 | 0.098 (-0.030, 0.226) | 0.137 | 0.294 | 0.049(-0.076,0.174) | 0.446 | 0.676 |
|  | Tertile2, CMD=0 | -0.046 (-0.124, 0.033) | 0.254 | 0.420 | 0.007(-0.08,0.093) | 0.883 | 0.924 |
|  | Tertile2, CMD=1 | 0.226 (0.093, 0.360) | 1.131e-03 | 0.033 | 0.172(0.037,0.306) | 1.396e-02 | 0.096 |
|  | Tertile2, CMD≥2 | 0.092 (-0.037, 0.222) | 0.166 | 0.324 | 0.222(0.073,0.37) | 4.492e-03 | 0.064 |
|  | Tertile3, CMD=0 | 0.117 (-0.020, 0.254) | 0.098 | 0.252 | 0.065(-0.056,0.185) | 0.296 | 0.576 |
|  | Tertile3, CMD=1 | 0.195 (0.058, 0.332) | 5.830e-03 | 0.046 | 0.298(0.141,0.454) | 3.039e-04 | 0.0219 |
|  | Tertile3, CMD≥2 | 0.325 (0.161, 0.488) | 1.831e-04 | 0.013 | 0.368(0.201,0.535) | 4.790e-05 | 0.0069 |
|  | **SNR** |  |  |  |  |  |  |
|  | Tertile1, CMD=0 | Ref |  |  | Ref |  |  |
|  | Tertile1, CMD=1 | 0.169 (0.063, 0.275) | 2.038e-03 | 0.033 | 0.178(0.049,0.308) | 8.207e-03 | 0.075 |
|  | Tertile1, CMD≥2 | 0.158 (0.039, 0.278) | 1.059e-02 | 0.066 | 0.137(-0.01,0.284) | 7.254e-02 | 0.255 |
|  | Tertile2, CMD=0 | -0.035 (-0.135, 0.066) | 0.502 | 0.657 | -0.038(-0.14,0.064) | 0.471 | 0.680 |
|  | Tertile2, CMD=1 | 0.178 (0.061, 0.295) | 3.441e-03 | 0.033 | 0.133(-0.005,0.271) | 6.074e-02 | 0.219 |
|  | Tertile2, CMD≥2 | 0.214 (0.077, 0.351) | 2.781e-03 | 0.033 | 0.148(-0.002,0.298) | 5.732e-02 | 0.212 |
|  | Tertile3, CMD=0 | 0.072 (-0.039, 0.183) | 0.204 | 0.363 | -0.006(-0.111,0.099) | 0.916 | 0.942 |
|  | Tertile3, CMD=1 | 0.161 (0.027, 0.296) | 2.005e-02 | 0.091 | 0.187(0.039,0.335) | 1.492e-02 | 0.098 |
|  | Tertile3, CMD≥2 | 0.235 (0.082, 0.388) | 3.315e-03 | 0.033 | 0.321(0.146,0.496) | 5.802e-04 | 0.028 |
| Delayed recall (by HVLT-R) | **Low_Frq** |  |  |  |  |  |  |
|  | Tertile1, CMD=0 | Ref |  |  | Ref |  |  |
|  | Tertile1, CMD=1 | -0.034(-0.153,0.085) | 0.576 | 0.709 | -0.045(-0.183,0.093) | 0.527 | 0.680 |
|  | Tertile1, CMD≥2 | 0.055(-0.113,0.224) | 0.520 | 0.669 | 0.185(-0.035,0.405) | 0.103 | 0.303 |
|  | Tertile2, CMD=0 | 0.013(-0.128,0.154) | 0.856 | 0.937 | -0.043(-0.192,0.105) | 0.571 | 0.713 |
|  | Tertile2, CMD=1 | 0.167(0.02,0.315) | 2.761e-02 | 0.110 | 0.089(-0.072,0.25) | 0.283 | 0.558 |
|  | Tertile2, CMD≥2 | 0.158(-0.015,0.331) | 0.077 | 0.222 | 0.069(-0.118,0.256) | 0.473 | 0.680 |
|  | Tertile3, CMD=0 | 0.232(0.052,0.412) | 1.326e-02 | 0.071 | 0.248(0.024,0.472) | 3.376e-02 | 0.154 |
|  | Tertile3, CMD=1 | 0.174(0.016,0.332) | 3.232e-02 | 0.126 | 0.125(-0.054,0.304) | 0.173 | 0.445 |
|  | Tertile3, CMD≥2 | 0.174(-0.036,0.384) | 0.107 | 0.257 | 0.099(-0.121,0.319) | 0.382 | 0.609 |
|  | **High_Frq** |  |  |  |  |  |  |
|  | Tertile1, CMD= 0 | Ref |  |  | Ref |  |  |
|  | Tertile1, CMD=1 | 0.024(-0.106,0.153) | 0.722 | 0.832 | 0.032(-0.115,0.18) | 0.666 | 0.773 |
|  | Tertile1, CMD≥2 | 0.012(-0.145,0.168) | 0.886 | 0.938 | -0.063(-0.255,0.129) | 0.519 | 0.680 |
|  | Tertile2, CMD=0 | 0.092(-0.056,0.24) | 0.226 | 0.387 | 0.005(-0.149,0.159) | 0.953 | 0.973 |
|  | Tertile2, CMD=1 | 0.07(-0.067,0.207) | 0.317 | 0.481 | 0.134(-0.023,0.29) | 0.097 | 0.297 |
|  | Tertile2, CMD≥2 | 0.2(0.029,0.371) | 2.405e-02 | 0.102 | 0.221(0.041,0.402) | 1.866e-02 | 0.117 |
|  | Tertile3, CMD=0 | 0.213(0.043,0.383) | 1.594e-02 | 0.0798 | 0.223(0.021,0.425) | 3.346e-02 | 0.154 |
|  | Tertile3, CMD=1 | 0.188(0.037,0.339) | 1.608e-02 | 0.0798 | 0.023(-0.135,0.181) | 0.777 | 0.848 |
|  | Tertile3, CMD≥2 | 0.235(0.04,0.43) | 2.003e-02 | 0.091 | 0.207(-0.001,0.415) | 5.474e-02 | 0.212 |
|  | **SNR** |  |  |  |  |  |  |
|  | Tertile1, CMD=0 | Ref |  |  | Ref |  |  |
|  | Tertile1, CMD=1 | 0.082(-0.024,0.187) | 0.132 | 0.292 | 0.058(-0.073,0.189) | 0.385 | 0.609 |
|  | Tertile1, CMD≥2 | 0.097(-0.036,0.231) | 0.156 | 0.321 | 0.083(-0.089,0.255) | 0.348 | 0.604 |
|  | Tertile2, CMD=0 | 0.125(-0.024,0.273) | 0.102 | 0.253 | 0.086(-0.077,0.248) | 0.306 | 0.580 |
|  | Tertile2, CMD=1 | 0.168(0.037,0.3) | 1.328e-02 | 0.071 | 0.049(-0.092,0.19) | 0.499 | 0.680 |
|  | Tertile2, CMD≥2 | 0.164(0.009,0.32) | 4.097e-02 | 0.137 | 0.133(-0.044,0.31) | 0.145 | 0.394 |
|  | Tertile3, CMD=0 | 0.24(0.093,0.387) | 1.790e-03 | 0.033 | 0.183(0.016,0.349) | 3.433e-02 | 0.154 |
|  | Tertile3, CMD=1 | 0.221(0.078,0.364) | 2.875e-03 | 0.033 | 0.19(0.024,0.356) | 2.685e-02 | 0.149 |
|  | Tertile3, CMD≥2 | 0.232(0.061,0.403) | 8.987e-03 | 0.059 | 0.226(0.037,0.415) | 2.193e-02 | 0.132 |
| Recognition (by HVLT-R) | **Low_Frq** |  |  |  |  |  |  |
|  | Tertile1, CMD=0 | Ref |  |  | Ref |  |  |
|  | Tertile1, CMD=1 | -0.063(-0.231,0.104) | 0.458 | 0.611 | -0.063(-0.251,0.124) | 0.510 | 0.680 |
|  | Tertile1, CMD≥2 | 0.062(-0.154,0.277) | 0.575 | 0.709 | 0.086(-0.181,0.354) | 0.529 | 0.680 |
|  | Tertile2, CMD=0 | -0.03(-0.218,0.158) | 0.754 | 0.842 | -0.014(-0.218,0.19) | 0.892 | 0.924 |
|  | Tertile2, CMD=1 | 0.051(-0.124,0.227) | 0.567 | 0.709 | 0.052(-0.142,0.246) | 0.599 | 0.713 |
|  | Tertile2, CMD≥2 | 0.155(-0.064,0.373) | 0.169 | 0.324 | 0.105(-0.129,0.338) | 0.382 | 0.609 |
|  | Tertile3, CMD=0 | -0.155(-0.37,0.06) | 0.162 | 0.324 | -0.225(-0.486,0.036) | 0.096 | 0.297 |
|  | Tertile3, CMD=1 | 0.063(-0.128,0.254) | 0.519 | 0.669 | -0.025(-0.243,0.193) | 0.820 | 0.888 |
|  | Tertile3, CMD≥2 | -0.133(-0.395,0.129) | 0.322 | 0.483 | 0.042(-0.248,0.332) | 0.777 | 0.848 |
|  | **High_Frq** |  |  |  |  |  |  |
|  | Tertile1, CMD=0 | Ref |  |  | Ref |  |  |
|  | Tertile1, CMD=1 | -0.113(-0.276,0.05) | 0.176 | 0.325 | -0.059(-0.238,0.121) | 0.524 | 0.680 |
|  | Tertile1, CMD≥2 | 0.001(-0.216,0.217) | 0.996 | 0.996 | 0.019(-0.237,0.275) | 0.887 | 0.924 |
|  | Tertile2, CMD=0 | -0.055(-0.237,0.126) | 0.551 | 0.696 | -0.005(-0.209,0.198) | 0.960 | 0.974 |
|  | Tertile2, CMD=1 | -0.005(-0.179,0.168) | 0.951 | 0.978 | -0.045(-0.246,0.155) | 0.657 | 0.769 |
|  | Tertile2, CMD≥2 | 0.119(-0.098,0.335) | 0.286 | 0.4576 | 0.157(-0.078,0.391) | 0.193 | 0.463 |
|  | Tertile3, CMD=0 | -0.185(-0.409,0.039) | 0.109 | 0.257 | 0.07(-0.182,0.323) | 0.587 | 0.713 |
|  | Tertile3, CMD=1 | 0.031(-0.151,0.213) | 0.739 | 0.838 | 0.097(-0.111,0.305) | 0.363 | 0.609 |
|  | Tertile3, CMD≥2 | -0.138(-0.382,0.106) | 0.270 | 0.437 | 0.135(-0.125,0.395) | 0.313 | 0.585 |
|  | **SNR** |  |  |  |  |  |  |
|  | Tertile1, CMD=0 | Ref |  |  | Ref |  |  |
|  | Tertile1, CMD=1 | 0.038(-0.107,0.184) | 0.608 | 0.726 | 0.052(-0.131,0.236) | 0.578 | 0.713 |
|  | Tertile1, CMD≥2 | 0.144(-0.046,0.334) | 0.141 | 0.299 | 0.018(-0.228,0.263) | 0.888 | 0.924 |
|  | Tertile2, CMD=0 | -0.018(-0.226,0.19) | 0.867 | 0.937 | -0.111(-0.339,0.118) | 0.346 | 0.604 |
|  | Tertile2, CMD=1 | 0.087(-0.078,0.251) | 0.305 | 0.472 | -0.107(-0.299,0.085) | 0.277 | 0.558 |
|  | Tertile2, CMD≥2 | 0.155(-0.065,0.376) | 0.171 | 0.324 | 0.159(-0.076,0.394) | 0.190 | 0.463 |
|  | Tertile3, CMD=0 | 0.067(-0.122,0.257) | 0.487 | 0.643 | 0.071(-0.137,0.278) | 0.506 | 0.680 |
|  | Tertile3, CMD=1 | 0.146(-0.042,0.335) | 0.131 | 0.292 | 0.051(-0.153,0.255) | 0.624 | 0.737 |
|  | Tertile3, CMD≥2 | 0.057(-0.178,0.292) | 0.634 | 0.742 | 0.096(-0.167,0.359) | 0.475 | 0.680 |
| Language (by BNT) | **Low_Frq** |  |  |  |  |  |  |
|  | Tertile1, CMD=0 | Ref |  |  | Ref |  |  |
|  | Tertile1, CMD=1 | 0.142(0.01,0.274) | 3.646e-02 | 0.135 | 0.12(-0.03,0.269) | 0.121 | 0.335 |
|  | Tertile1, CMD≥2 | 0.216(0.05,0.381) | 1.256e-02 | 0.071 | 0.162(-0.039,0.364) | 0.120 | 0.335 |
|  | Tertile2, CMD=0 | 0.103(-0.04,0.245) | 0.160 | 0.324 | 0.069(-0.084,0.223) | 0.377 | 0.609 |
|  | Tertile2, CMD=1 | 0.059(-0.078,0.195) | 0.399 | 0.558 | 0.063(-0.083,0.21) | 0.399 | 0.625 |
|  | Tertile2, CMD≥2 | 0.276(0.101,0.451) | 2.638E-03 | 0.033 | 0.272(0.075,0.469) | 8.381E-03 | 0.075 |
|  | Tertile3, CMD=0 | 0.243(0.072,0.413) | 6.349E-03 | 0.046 | 0.126(-0.079,0.331) | 0.232 | 0.499 |
|  | Tertile3, CMD=1 | 0.129(-0.024,0.283) | 0.101 | 0.253 | 0.049(-0.134,0.233) | 0.599 | 0.713 |
|  | Tertile3, CMD≥2 | 0.128(-0.082,0.337) | 0.236 | 0.340 | 0.194(-0.024,0.412) | 0.085 | 0.289 |
|  | **High_Frq** |  |  |  |  |  |  |
|  | Tertile1, CMD=0 | Ref |  |  | Ref |  |  |
|  | Tertile1, CMD=1 | 0.009(-0.136,0.154) | 0.908 | 0.954 | 0.054(-0.114,0.222) | 0.529 | 0.680 |
|  | Tertile1, CMD≥2 | 0.097(-0.096,0.29) | 0.329 | 0.488 | 0.065(-0.175,0.304) | 0.598 | 0.713 |
|  | Tertile2, CMD=0 | -0.066(-0.216,0.083) | 0.388 | 0.548 | -0.083(-0.253,0.087) | 0.340 | 0.604 |
|  | Tertile2, CMD=1 | -0.004(-0.148,0.141) | 0.962 | 0.979 | -0.018(-0.177,0.142) | 0.829 | 0.891 |
|  | Tertile2, CMD≥2 | 0.141(-0.051,0.334) | 0.153 | 0.319 | 0.21(0.004,0.416) | 4.866e-02 | 0.201 |
|  | Tertile3, CMD=0 | 0.05(-0.139,0.239) | 0.607 | 0.726 | -0.038(-0.242,0.166) | 0.717 | 0.819 |
|  | Tertile3, CMD=1 | 0.016(-0.144,0.177) | 0.842 | 0.933 | -0.077(-0.263,0.109) | 0.420 | 0.650 |
|  | Tertile3, CMD≥2 | 0.018(-0.203,0.238) | 0.877 | 0.937 | 0.017(-0.219,0.252) | 0.889 | 0.924 |
|  | **SNR** |  |  |  |  |  |  |
|  | Tertile1, CMD=0 | Ref |  |  | Ref |  |  |
|  | Tertile1, CMD=1 | 0.054(-0.059,0.167) | 0.350 | 0.509 | 0.103(-0.042,0.248) | 0.166 | 0.435 |
|  | Tertile1, CMD≥2 | 0.136(-0.018,0.291) | 0.086 | 0.238 | 0.143(-0.052,0.338) | 0.156 | 0.416 |
|  | Tertile2, CMD=0 | 0.078(-0.082,0.238) | 0.344 | 0.505 | 0.092(-0.091,0.276) | 0.327 | 0.596 |
|  | Tertile2, CMD=1 | 0.104(-0.032,0.24) | 0.136 | 0.294 | 0.051(-0.106,0.207) | 0.527 | 0.680 |
|  | Tertile2, CMD≥2 | 0.151(-0.026,0.328) | 0.097 | 0.252 | 0.066(-0.119,0.25) | 0.488 | 0.680 |
|  | Tertile3, CMD=0 | 0.163(0.007,0.319) | 4.252e-02 | 0.137 | 0.046(-0.111,0.203) | 0.569 | 0.713 |
|  | Tertile3, CMD=1 | 0.067(-0.09,0.224) | 0.403 | 0.558 | 0.055(-0.111,0.221) | 0.519 | 0.680 |
|  | Tertile3, CMD≥2 | 0.226(0.029,0.423) | 2.628e-02 | 0.108 | 0.327(0.108,0.546) | 4.591e-03 | 0.064 |
| Language (by animal fluency) | **Low_Frq** |  |  |  |  |  |  |
|  | Tertile1, CMD=0 | Ref |  |  | Ref |  |  |
|  | Tertile1, CMD=1 | 0.217(0.075,0.359) | 3.257e-03 | 0.033 | 0.227(0.073,0.382) | 4.895e-03 | 0.064 |
|  | Tertile1, CMD≥2 | 0.144(-0.022,0.309) | 0.093 | 0.251 | 0.117(-0.079,0.313) | 0.246 | 0.521 |
|  | Tertile2, CMD=0 | 0.077(-0.067,0.22) | 0.298 | 0.466 | 0.098(-0.058,0.254) | 0.223 | 0.499 |
|  | Tertile2, CMD=1 | 0.218(0.072,0.365) | 4.112e-03 | 0.035 | 0.286(0.118,0.453) | 1.117e-03 | 0.032 |
|  | Tertile2, CMD≥2 | 0.297(0.117,0.478) | 1.727e-03 | 0.033 | 0.274(0.083,0.465) | 6.275e-03 | 0.065 |
|  | Tertile3, CMD=0 | 0.157(-0.015,0.328) | 0.077 | 0.222 | 0.04(-0.15,0.231) | 0.680 | 0.783 |
|  | Tertile3, CMD=1 | 0.165(0.005,0.324) | 4.463E-02 | 0.140 | 0.108(-0.061,0.276) | 0.213 | 0.495 |
|  | Tertile3, CMD≥2 | 0.005(-0.199,0.209) | 0.965 | 0.979 | 0.256(0.03,0.481) | 2.973E-02 | 0.149 |
|  | **High_Frq** |  |  |  |  |  |  |
|  | Tertile1, CMD=0 | Ref |  |  | Ref |  |  |
|  | Tertile1, CMD=1 | 0.186(0.044,0.329) | 1.138e-02 | 0.068 | 0.182(0.02,0.344) | 0.030 | 0.149 |
|  | Tertile1, CMD≥2 | 0.219(0.036,0.401) | 2.084e-02 | 0.091 | 0.101(-0.125,0.326) | 0.384 | 0.609 |
|  | Tertile2, CMD=0 | 0.024(-0.121,0.169) | 0.745 | 0.838 | -0.097(-0.253,0.059) | 0.229 | 0.499 |
|  | Tertile2, CMD=1 | 0.235(0.083,0.388) | 2.946e-03 | 0.033 | 0.228(0.052,0.403) | 1.252e-02 | 0.092 |
|  | Tertile2, CMD≥2 | 0.121(-0.052,0.294) | 0.174 | 0.325 | 0.254(0.059,0.45) | 1.282e-02 | 0.092 |
|  | Tertile3, CMD=0 | 0.192(0.015,0.369) | 3.549e-02 | 0.134 | 0.174(-0.022,0.37) | 8.635e-02 | 0.289 |
|  | Tertile3, CMD=1 | 0.137(-0.018,0.292) | 0.086 | 0.238 | 0.063(-0.116,0.243) | 0.490 | 0.680 |
|  | Tertile3, CMD≥2 | 0.117(-0.089,0.323) | 0.269 | 0.437 | 0.128(-0.102,0.358) | 0.279 | 0.558 |
|  | **SNR** |  |  |  |  |  |  |
|  | Tertile1, CMD=0 | Ref |  |  | Ref |  |  |
|  | Tertile1, CMD=1 | 0.178(0.052,0.304) | 6.064e-03 | 0.046 | 0.232(0.07,0.394) | 6.005e-03 | 0.0656 |
|  | Tertile1, CMD≥2 | 0.257(0.099,0.414) | 1.830e-03 | 0.0336 | 0.204(0.003,0.405) | 4.998e-02 | 0.201 |
|  | Tertile2, CMD=0 | -0.012(-0.17,0.146) | 0.878 | 0.937 | -0.105(-0.276,0.065) | 0.229 | 0.499 |
|  | Tertile2, CMD=1 | 0.165(0.027,0.304) | 2.062e-02 | 0.091 | 0.089(-0.08,0.257) | 0.305 | 0.580 |
|  | Tertile2, CMD≥2 | 0.041(-0.131,0.214) | 0.639 | 0.742 | 0.132(-0.07,0.334) | 0.205 | 0.484 |
|  | Tertile3, CMD=0 | 0.105(-0.05,0.259) | 0.186 | 0.338 | -0.001(-0.17,0.168) | 0.993 | 0.993 |
|  | Tertile3, CMD=1 | 0.168(0.011,0.326) | 3.748E-02 | 0.135 | 0.067(-0.099,0.234) | 0.431 | 0.660 |
|  | Tertile3, CMD≥2 | 0.101(-0.092,0.293) | 0.308 | 0.472 | 0.125(-0.095,0.344) | 0.271 | 0.557 |
