# Supplementary material for: Peripheral and central auditory dysfunction, cardiometabolic multimorbidity, and cognitive performance in community-dwelling older adults: a cross-sectional study
Source: Front Neurosci. 2026 Jan 16;19:1646313. doi: 10.3389/fnins.2025.1646313 (PMC12856757; doi:10.3389/fnins.2025.1646313)
Supplement: Supplementary file 9 [file Table_8.docx]

Supplementary Table 7. The joint effects of LPTA, HPTA, or SNR and CMD stratification on domain-specific cognitive performance in Model 2

| Groups |  | Total sample Model 2 | | | Sensitivity test Model 2 | | |
| --- | --- | --- | --- | --- | --- | --- | --- |
|  |  | β （95%CI） | P value | Adjusted P value | β（95%CI） | P value | Adjusted P value |
| Processing by TMT A | **Low_Frq** |  |  |  |  |  |  |
|  | Tertile1, CMD=0 | Ref |  |  | Ref |  |  |
|  | Tertile1, CMD=1 | -0.018(-0.141,0.105) | 0.774 | 0.938 | 0.001(-0.131,0.133) | 0.985 | 0.997 |
|  | Tertile1, CMD ≥2 | 0.061(-0.123,0.245) | 0.520 | 0.832 | 0.161(-0.042,0.363) | 0.127 | 0.419 |
|  | Tertile2, CMD=0 | -0.012(-0.162,0.138) | 0.878 | 0.951 | 0.065(-0.109,0.239) | 0.465 | 0.752 |
|  | Tertile2, CMD=1 | 0.013(-0.149,0.174) | 0.878 | 0.951 | 0.109(-0.053,0.27) | 0.189 | 0.534 |
|  | Tertile2, CMD ≥2 | 0.1(-0.129,0.329) | 0.394 | 0.731 | 0.09(-0.115,0.295) | 0.391 | 0.7038 |
|  | Tertile3, CMD=0 | -0.068(-0.349,0.213) | 0.636 | 0.876 | -0.047(-0.276,0.183) | 0.690 | 0.887 |
|  | Tertile3, CMD=1 | 0.024(-0.197,0.245) | 0.831 | 0.938 | 0.01(-0.188,0.207) | 0.925 | 0.99 |
|  | Tertile3, CMD ≥2 | 0.179(-0.14,0.497) | 0.275 | 0.612 | 0.29(-0.013,0.593) | 6.631e-02 | 0.345 |
|  | **High_Frq** |  |  |  |  |  |  |
|  | Tertile1, CMD= 0 | Ref |  |  | Ref |  |  |
|  | Tertile1, CMD=1 | -0.021(-0.147,0.105) | 0.743 | 0.934 | 0.033(-0.103,0.169) | 0.636 | 0.882 |
|  | Tertile1, CMD ≥2 | 0.035(-0.143,0.214) | 0.700 | 0.892 | 0.162(-0.045,0.369) | 0.132 | 0.422 |
|  | Tertile2, CMD=0 | 0.006(-0.145,0.157) | 0.936 | 0.977 | 0.09(-0.067,0.246) | 0.267 | 0.574 |
|  | Tertile2, CMD=1 | 0.01(-0.144,0.164) | 0.901 | 0.961 | 0.035(-0.113,0.183) | 0.646 | 0.882 |
|  | Tertile2, CMD ≥2 | 0.06(-0.163,0.282) | 0.599 | 0.871 | 0.182(-0.003,0.366) | 5.796e-02 | 0.345 |
|  | Tertile3, CMD=0 | 0.033(-0.269,0.336) | 0.829 | 0.938 | 0.049(-0.187,0.285) | 0.684 | 0.887 |
|  | Tertile3, CMD=1 | 0.048(-0.157,0.253) | 0.647 | 0.879 | 0.151(-0.054,0.356) | 0.152 | 0.456 |
|  | Tertile3, CMD ≥2 | 0.47(0.18,0.76) | 2.129e-03 | 0.102 | 0.295(0.028,0.562) | 3.410e-02 | 0.307 |
|  | **SNR** |  |  |  |  |  |  |
|  | Tertile1, CMD=0 | Ref |  |  | Ref |  |  |
|  | Tertile1, CMD=1 | 0.012(-0.109,0.134) | 0.843 | 0.938 | 0.026(-0.131,0.183) | 0.745 | 0.914 |
|  | Tertile1, CMD ≥2 | 0.116(-0.054,0.286) | 0.183 | 0.527 | 0.095(-0.112,0.303) | 0.372 | 0.690 |
|  | Tertile2, CMD=0 | -0.089(-0.258,0.08) | 0.302 | 0.621 | -0.042(-0.212,0.127) | 0.627 | 0.882 |
|  | Tertile2, CMD=1 | -0.018(-0.144,0.109) | 0.785 | 0.938 | -0.044(-0.202,0.114) | 0.590 | 0.868 |
|  | Tertile2, CMD ≥2 | 0.102(-0.094,0.299) | 0.310 | 0.629 | 0.022(-0.211,0.255) | 0.854 | 0.968 |
|  | Tertile3, CMD=0 | -0.015(-0.19,0.16) | 0.870 | 0.951 | -0.112(-0.304,0.08) | 0.257 | 0.574 |
|  | Tertile3, CMD=1 | 0.078(-0.104,0.26) | 0.401 | 0.731 | -0.031(-0.2,0.138) | 0.720 | 0.904 |
|  | Tertile3, CMD ≥2 | 0.127(-0.077,0.331) | 0.226 | 0.576 | 0.14(-0.102,0.383) | 0.262 | 0.574 |
| Attention/executive function (by TMT B) | **Low_Frq** |  |  |  |  |  |  |
|  | Tertile1, CMD=0 | Ref |  |  | Ref |  |  |
|  | Tertile1, CMD=1 | 0.133(0.008,0.258) | 3.872e-02 | 0.242 | 0.12(-0.02,0.261) | 0.098 | 0.398 |
|  | Tertile1, CMD ≥2 | 0.088(-0.055,0.23) | 0.232 | 0.576 | 0.241(0.069,0.413) | 8.154e-03 | 0.168 |
|  | Tertile2, CMD=0 | -0.01(-0.107,0.088) | 0.847 | 0.938 | -0.007(-0.128,0.114) | 0.907 | 0.989 |
|  | Tertile2, CMD=1 | 0.136(-0.015,0.288) | 8.083e-02 | 0.349 | 0.201(0.045,0.357) | 1.302e-02 | 0.202 |
|  | Tertile2, CMD ≥2 | 0.172(-0.021,0.364) | 8.483e-02 | 0.349 | 0.051(-0.105,0.207) | 0.521 | 0.808 |
|  | Tertile3, CMD=0 | 0.059(-0.16,0.278) | 0.597 | 0.871 | -0.031(-0.172,0.109) | 0.666 | 0.887 |
|  | Tertile3, CMD=1 | 0.124(-0.074,0.322) | 0.220 | 0.576 | 0.047(-0.155,0.249) | 0.649 | 0.882 |
|  | Tertile3, CMD ≥2 | 0.024(-0.188,0.236) | 0.825 | 0.938 | 0.275(0.046,0.504) | 2.248e-02 | 0.231 |
|  | **High_Frq** |  |  |  |  |  |  |
|  | Tertile1, CMD= 0 | Ref |  |  | Ref |  |  |
|  | Tertile1, CMD=1 | 0.056(-0.046,0.158) | 0.285 | 0.612 | 0.084(-0.028,0.197) | 0.146 | 0.456 |
|  | Tertile1, CMD ≥2 | 0.075(-0.066,0.215) | 0.301 | 0.621 | 0.109(-0.038,0.256) | 0.152 | 0.456 |
|  | Tertile2, CMD=0 | -0.033(-0.126,0.06) | 0.490 | 0.832 | 0.003(-0.094,0.101) | 0.950 | 0.995 |
|  | Tertile2, CMD=1 | 0.21(0.053,0.366) | 9.617e-03 | 0.155 | 0.182(0.039,0.324) | 1.402e-02 | 0.202 |
|  | Tertile2, CMD ≥2 | 0.109(-0.052,0.269) | 0.189 | 0.534 | 0.188(0.013,0.363) | 3.944e-02 | 0.334 |
|  | Tertile3, CMD=0 | 0.082(-0.155,0.319) | 0.501 | 0.832 | 0.079(-0.08,0.237) | 0.334 | 0.659 |
|  | Tertile3, CMD=1 | 0.202(0.013,0.391) | 3.769e-02 | 0.242 | 0.402(0.182,0.623) | 5.349e-04 | 0.0775 |
|  | Tertile3, CMD ≥2 | 0.461(0.245,0.677) | 7.384e-05 | 0.011 | 0.318(0.097,0.539) | 6.562e-03 | 0.168 |
|  | **SNR** |  |  |  |  |  |  |
|  | Tertile1, CMD=0 | Ref |  |  | Ref |  |  |
|  | Tertile1, CMD=1 | 0.141(0.03,0.252) | 1.395e-02 | 0.155 | 0.178(0.042,0.315) | 1.217e-02 | 0.202 |
|  | Tertile1, CMD ≥2 | 0.153(0.018,0.289) | 2.901e-02 | 0.232 | 0.154(-0.006,0.313) | 6.432e-02 | 0.345 |
|  | Tertile2, CMD=0 | -0.025(-0.143,0.094) | 0.683 | 0.892 | 0.002(-0.099,0.104) | 0.965 | 0.995 |
|  | Tertile2, CMD=1 | 0.107(-0.019,0.232) | 0.097 | 0.378 | 0.107(-0.047,0.261) | 0.178 | 0.513 |
|  | Tertile2, CMD ≥2 | 0.194(0.029,0.358) | 2.337e-02 | 0.220 | 0.167(-0.005,0.339) | 6.159e-02 | 0.345 |
|  | Tertile3, CMD=0 | 0.033(-0.104,0.171) | 0.633 | 0.876 | 0.015(-0.106,0.135) | 0.809 | 0.938 |
|  | Tertile3, CMD=1 | 0.145(-0.016,0.307) | 0.080 | 0.349 | 0.146(-0.013,0.304) | 7.421e-02 | 0.345 |
|  | Tertile3, CMD ≥2 | 0.183(0.014,0.353) | 3.668e-02 | 0.242 | 0.342(0.139,0.545) | 1.662e-03 | 0.0798 |
| Delayed recall (by HVLT-R) | **Low_Frq** |  |  |  |  |  |  |
|  | Tertile1, CMD=0 | Ref |  |  | Ref |  |  |
|  | Tertile1, CMD=1 | 0.031(-0.099,0.162) | 0.639 | 0.876 | -0.038(-0.2,0.123) | 0.645 | 0.882 |
|  | Tertile1, CMD ≥2 | 0.107(-0.089,0.303) | 0.288 | 0.612 | 0.227(-0.043,0.497) | 0.105 | 0.398 |
|  | Tertile2, CMD=0 | 0.006(-0.159,0.17) | 0.945 | 0.979 | -0.075(-0.255,0.105) | 0.418 | 0.709 |
|  | Tertile2, CMD=1 | 0.175(-0.008,0.357) | 6.305e-02 | 0.313 | 0.103(-0.085,0.292) | 0.286 | 0.588 |
|  | Tertile2, CMD ≥2 | 0.249(-0.009,0.506) | 6.192e-02 | 0.313 | 0.03(-0.219,0.278) | 0.814 | 0.938 |
|  | Tertile3, CMD=0 | 0.288(-0.026,0.601) | 7.550e-02 | 0.349 | 0.209(-0.122,0.539) | 0.221 | 0.562 |
|  | Tertile3, CMD=1 | 0.1(-0.132,0.332) | 0.401 | 0.731 | 0.217(-0.041,0.474) | 0.102 | 0.398 |
|  | Tertile3, CMD ≥2 | 0.139(-0.18,0.458) | 0.395 | 0.731 | 0.137(-0.21,0.484) | 0.442 | 0.740 |
|  | **High_Frq** |  |  |  |  |  |  |
|  | Tertile1, CMD= 0 | Ref |  |  | Ref |  |  |
|  | Tertile1, CMD=1 | 0.049(-0.094,0.192) | 0.502 | 0.832 | 0.069(-0.096,0.234) | 0.415 | 0.708 |
|  | Tertile1, CMD ≥2 | 0.019(-0.17,0.209) | 0.842 | 0.938 | 0(-0.238,0.238) | 0.998 | 0.998 |
|  | Tertile2, CMD=0 | -0.023(-0.203,0.158) | 0.806 | 0.938 | -0.024(-0.221,0.173) | 0.812 | 0.938 |
|  | Tertile2, CMD=1 | -0.011(-0.179,0.158) | 0.900 | 0.961 | 0.106(-0.079,0.29) | 0.265 | 0.574 |
|  | Tertile2, CMD ≥2 | 0.169(-0.057,0.394) | 0.146 | 0.467 | 0.243(0.008,0.479) | 4.720e-02 | 0.345 |
|  | Tertile3, CMD=0 | 0.133(-0.163,0.429) | 0.382 | 0.731 | 0.081(-0.181,0.343) | 0.548 | 0.831 |
|  | Tertile3, CMD=1 | 0.069(-0.135,0.273) | 0.510 | 0.832 | 0.016(-0.212,0.245) | 0.890 | 0.986 |
|  | Tertile3, CMD ≥2 | 0.215(-0.081,0.512) | 0.159 | 0.486 | 0.381(0.067,0.694) | 2.072e-02 | 0.229 |
|  | **SNR** |  |  |  |  |  |  |
|  | Tertile1, CMD=0 | Ref |  |  | Ref |  |  |
|  | Tertile1, CMD=1 | 0.091(-0.026,0.207) | 0.131 | 0.449 | 0.037(-0.102,0.177) | 0.599 | 0.868 |
|  | Tertile1, CMD ≥2 | 0.1(-0.061,0.261) | 0.225 | 0.576 | 0.074(-0.119,0.267) | 0.455 | 0.745 |
|  | Tertile2, CMD=0 | 0.104(-0.076,0.284) | 0.261 | 0.612 | 0.086(-0.092,0.264) | 0.345 | 0.662 |
|  | Tertile2, CMD=1 | 0.194(0.049,0.339) | 9.811e-03 | 0.155 | 0.024(-0.145,0.192) | 0.784 | 0.929 |
|  | Tertile2, CMD ≥2 | 0.142(-0.048,0.332) | 0.146 | 0.467 | 0.099(-0.118,0.315) | 0.374 | 0.690 |
|  | Tertile3, CMD=0 | 0.147(-0.03,0.324) | 0.106 | 0.382 | 0.102(-0.1,0.305) | 0.325 | 0.650 |
|  | Tertile3, CMD=1 | 0.145(-0.029,0.319) | 0.106 | 0.382 | 0.203(0.022,0.384) | 3.026e-02 | 0.290 |
|  | Tertile3, CMD ≥2 | 0.223(0.015,0.431) | 3.809e-02 | 0.242 | 0.323(0.065,0.58) | 1.694e-02 | 0.204 |
| Recognition (by HVLT-R) | **Low_Frq** |  |  |  |  |  |  |
|  | Tertile1, CMD=0 | Ref |  |  | Ref |  |  |
|  | Tertile1, CMD=1 | -0.037(-0.199,0.126) | 0.659 | 0.887 | -0.071(-0.237,0.095) | 0.403 | 0.706 |
|  | Tertile1, CMD ≥2 | -0.029(-0.25,0.192) | 0.796 | 0.938 | 0.036(-0.222,0.294) | 0.787 | 0.929 |
|  | Tertile2, CMD=0 | -0.003(-0.197,0.191) | 0.975 | 0.990 | -0.055(-0.278,0.168) | 0.632 | 0.882 |
|  | Tertile2, CMD=1 | 0.011(-0.185,0.207) | 0.913 | 0.967 | -0.071(-0.287,0.145) | 0.522 | 0.808 |
|  | Tertile2, CMD ≥2 | 0.088(-0.195,0.371) | 0.544 | 0.851 | -0.123(-0.402,0.156) | 0.391 | 0.704 |
|  | Tertile3, CMD=0 | 0.016(-0.31,0.342) | 0.922 | 0.969 | -0.291(-0.595,0.014) | 6.660e-02 | 0.345 |
|  | Tertile3, CMD=1 | 0.062(-0.183,0.307) | 0.622 | 0.876 | 0.003(-0.282,0.288) | 0.982 | 0.997 |
|  | Tertile3, CMD ≥2 | 0.006(-0.372,0.384) | 0.975 | 0.990 | -0.06(-0.386,0.266) | 0.719 | 0.904 |
|  | **High_Frq** |  |  |  |  |  |  |
|  | Tertile1, CMD=0 | Ref |  |  | Ref |  |  |
|  | Tertile1, CMD=1 | -0.082(-0.227,0.062) | 0.267 | 0.612 | -0.061(-0.217,0.096) | 0.449 | 0.743 |
|  | Tertile1, CMD ≥2 | -0.163(-0.385,0.059) | 0.154 | 0.482 | -0.145(-0.401,0.112) | 0.274 | 0.580 |
|  | Tertile2, CMD=0 | -0.002(-0.199,0.196) | 0.988 | 0.995 | 0.057(-0.153,0.266) | 0.597 | 0.86832 |
|  | Tertile2, CMD=1 | -0.025(-0.221,0.172) | 0.806 | 0.938 | -0.032(-0.239,0.175) | 0.763 | 0.923 |
|  | Tertile2, CMD ≥2 | 0.066(-0.187,0.318) | 0.611 | 0.874 | 0.055(-0.195,0.306) | 0.666 | 0.887 |
|  | Tertile3, CMD=0 | -0.206(-0.534,0.123) | 0.224 | 0.576 | 0.029(-0.259,0.318) | 0.843 | 0.963 |
|  | Tertile3, CMD=1 | 0.074(-0.155,0.304) | 0.526 | 0.832 | 0.019(-0.253,0.292) | 0.890 | 0.986 |
|  | Tertile3, CMD ≥2 | -0.095(-0.418,0.229) | 0.568 | 0.870 | 0.2(-0.129,0.529) | 0.238 | 0.562 |
|  | **SNR** |  |  |  |  |  |  |
|  | Tertile1, CMD=0 | Ref |  |  | Ref |  |  |
|  | Tertile1, CMD=1 | 0.053(-0.094,0.201) | 0.480 | 0.832 | 0.028(-0.157,0.213) | 0.770 | 0.924 |
|  | Tertile1, CMD ≥2 | 0.023(-0.191,0.236) | 0.837 | 0.938 | -0.094(-0.352,0.163) | 0.476 | 0.761 |
|  | Tertile2, CMD=0 | 0.088(-0.142,0.318) | 0.457 | 0.812 | -0.045(-0.256,0.166) | 0.675 | 0.887 |
|  | Tertile2, CMD=1 | 0.083(-0.087,0.253) | 0.339 | 0.678 | -0.001(-0.204,0.202) | 0.990 | 0.997 |
|  | Tertile2, CMD ≥2 | 0.05(-0.197,0.298) | 0.693 | 0.892 | 0.022(-0.254,0.297) | 0.878 | 0.986 |
|  | Tertile3, CMD=0 | 0.198(-0.004,0.4) | 5.746e-02 | 0.313 | 0.214(-0.009,0.437) | 6.397e-02 | 0.345 |
|  | Tertile3, CMD=1 | 0.26(0.058,0.461) | 1.282e-02 | 0.155 | 0.045(-0.167,0.257) | 0.679 | 0.887 |
|  | Tertile3, CMD ≥2 | 0.051(-0.209,0.311) | 0.700 | 0.892 | 0.128(-0.173,0.429) | 0.407 | 0.706 |
| Language (by BNT) | **Low_Frq** |  |  |  |  |  |  |
|  | Tertile1, CMD=0 | Ref |  |  | Ref |  |  |
|  | Tertile1, CMD=1 | 0.128(-0.02,0.277) | 0.093 | 0.372 | 0.061(-0.109,0.231) | 0.481 | 0.761 |
|  | Tertile1, CMD ≥2 | 0.213(0.019,0.407) | 3.476e-02 | 0.242 | 0.203(-0.033,0.438) | 0.097 | 0.398 |
|  | Tertile2, CMD=0 | 0.158(-0.007,0.323) | 6.311e-02 | 0.313 | 0.182(-0.005,0.369) | 6.077e-02 | 0.345 |
|  | Tertile2, CMD=1 | 0.022(-0.147,0.191) | 0.798 | 0.938 | 0.076(-0.1,0.252) | 0.398 | 0.706 |
|  | Tertile2, CMD ≥2 | 0.338(0.084,0.592) | 1.100e-02 | 0.155 | 0.203(-0.055,0.461) | 0.128 | 0.419 |
|  | Tertile3, CMD=0 | 0.279(-0.001,0.56) | 5.423e-02 | 0.313 | 0.128(-0.152,0.409) | 0.373 | 0.690 |
|  | Tertile3, CMD=1 | 0.193(-0.036,0.422) | 0.102 | 0.382 | 0.006(-0.254,0.266) | 0.963 | 0.995 |
|  | Tertile3, CMD ≥2 | 0.1(-0.208,0.409) | 0.525 | 0.832 | 0.248(-0.055,0.551) | 0.114 | 0.398 |
|  | **High_Frq** |  |  |  |  |  |  |
|  | Tertile1, CMD=0 | Ref |  |  | Ref |  |  |
|  | Tertile1, CMD=1 | -0.024(-0.181,0.133) | 0.763 | 0.938 | -0.005(-0.185,0.174) | 0.954 | 0.995 |
|  | Tertile1, CMD ≥2 | 0.031(-0.197,0.258) | 0.793 | 0.938 | 0.015(-0.273,0.302) | 0.921 | 0.994 |
|  | Tertile2, CMD=0 | -0.172(-0.346,0.002) | 0.056 | 0.313 | -0.105(-0.309,0.1) | 0.320 | 0.649 |
|  | Tertile2, CMD=1 | -0.124(-0.297,0.049) | 0.162 | 0.486 | -0.112(-0.295,0.071) | 0.234 | 0.5618 |
|  | Tertile2, CMD ≥2 | 0.07(-0.182,0.322) | 0.588 | 0.871 | 0.073(-0.185,0.331) | 0.581 | 0.868 |
|  | Tertile3, CMD=0 | -0.149(-0.469,0.172) | 0.366 | 0.712 | -0.167(-0.431,0.098) | 0.221 | 0.5618 |
|  | Tertile3, CMD=1 | -0.046(-0.267,0.175) | 0.686 | 0.892 | -0.16(-0.418,0.097) | 0.225 | 0.5618 |
|  | Tertile3, CMD ≥2 | -0.088(-0.413,0.237) | 0.597 | 0.871 | 0.093(-0.256,0.443) | 0.603 | 0.868 |
|  | **SNR** |  |  |  |  |  |  |
|  | Tertile1, CMD=0 | Ref |  |  | Ref |  |  |
|  | Tertile1, CMD=1 | 0.083(-0.042,0.208) | 0.195 | 0.540 | 0.137(-0.023,0.297) | 0.097 | 0.398 |
|  | Tertile1, CMD ≥2 | 0.094(-0.079,0.268) | 0.289 | 0.612 | 0.132(-0.081,0.344) | 0.230 | 0.562 |
|  | Tertile2, CMD=0 | 0.103(-0.086,0.292) | 0.289 | 0.612 | 0.129(-0.07,0.329) | 0.209 | 0.562 |
|  | Tertile2, CMD=1 | 0.1(-0.042,0.242) | 0.171 | 0.503 | 0.03(-0.151,0.21) | 0.749 | 0.914 |
|  | Tertile2, CMD ≥2 | 0.195(-0.018,0.408) | 7.674e-02 | 0.349 | 0.007(-0.208,0.222) | 0.949 | 0.995 |
|  | Tertile3, CMD=0 | 0.06(-0.125,0.246) | 0.523 | 0.832 | -0.012(-0.194,0.171) | 0.900 | 0.9898 |
|  | Tertile3, CMD=1 | 0.031(-0.158,0.221) | 0.746 | 0.934 | 0.089(-0.094,0.272) | 0.343 | 0.66284 |
|  | Tertile3, CMD ≥2 | 0.302(0.084,0.52) | 7.900e-03 | 0.155 | 0.473(0.194,0.751) | 1.528e-03 | 0.0798 |
| Language (by animal fluency) | **Low_Frq** |  |  |  |  |  |  |
|  | Tertile1, CMD=0 | Ref |  |  | Ref |  |  |
|  | Tertile1, CMD=1 | 0.205(0.047,0.363) | 1.227e-02 | 0.155 | 0.248(0.071,0.425) | 7.179e-03 | 0.168 |
|  | Tertile1, CMD ≥2 | 0.106(-0.082,0.295) | 0.271 | 0.612 | 0.141(-0.08,0.361) | 0.217 | 0.562 |
|  | Tertile2, CMD=0 | 0.096(-0.06,0.252) | 0.232 | 0.576 | 0.11(-0.07,0.29) | 0.235 | 0.562 |
|  | Tertile2, CMD=1 | 0.258(0.076,0.439) | 6.195e-03 | 0.155 | 0.23(0.044,0.416) | 1.704e-02 | 0.204 |
|  | Tertile2, CMD ≥2 | 0.302(0.045,0.558) | 2.378e-02 | 0.220 | 0.159(-0.086,0.403) | 0.208 | 0.562 |
|  | Tertile3, CMD=0 | 0.169(-0.123,0.461) | 0.260 | 0.612 | 0.087(-0.182,0.355) | 0.530 | 0.812 |
|  | Tertile3, CMD=1 | 0.298(0.065,0.532) | 1.358e-02 | 0.155 | 0.196(-0.04,0.432) | 0.107 | 0.398 |
|  | Tertile3, CMD ≥2 | -0.001(-0.318,0.316) | 0.996 | 0.996 | 0.292(-0.021,0.604) | 7.316e-02 | 0.345 |
|  | **High_Frq** |  |  |  |  |  |  |
|  | Tertile1, CMD=0 | Ref |  |  | Ref |  |  |
|  | Tertile1, CMD=1 | 0.131(-0.017,0.279) | 8.490e-02 | 0.349 | 0.099(-0.07,0.269) | 0.254 | 0.574 |
|  | Tertile1, CMD ≥2 | 0.07(-0.125,0.265) | 0.484 | 0.832 | -0.005(-0.266,0.255) | 0.967 | 0.995 |
|  | Tertile2, CMD=0 | 0.045(-0.123,0.213) | 0.598 | 0.871 | -0.102(-0.277,0.073) | 0.257 | 0.574 |
|  | Tertile2, CMD=1 | 0.253(0.07,0.436) | 7.765e-03 | 0.155 | 0.165(-0.039,0.369) | 0.116 | 0.398 |
|  | Tertile2, CMD ≥2 | 0.129(-0.097,0.355) | 0.266 | 0.612 | 0.228(-0.006,0.463) | 6.085e-02 | 0.345 |
|  | Tertile3, CMD=0 | 0.063(-0.238,0.364) | 0.682 | 0.892 | 0.26(0.009,0.511) | 4.624e-02 | 0.345 |
|  | Tertile3, CMD=1 | 0.174(-0.045,0.393) | 0.121 | 0.425 | 0.235(-0.02,0.49) | 7.361e-02 | 0.345 |
|  | Tertile3, CMD ≥2 | 0.115(-0.172,0.402) | 0.435 | 0.783 | 0.179(-0.144,0.503) | 0.282 | 0.588 |
|  | **SNR** |  |  |  |  |  |  |
|  | Tertile1, CMD=0 | Ref |  |  | Ref |  |  |
|  | Tertile1, CMD=1 | 0.234(0.098,0.37) | 9.054e-04 | 0.065 | 0.262(0.086,0.437) | 4.414e-03 | 0.159 |
|  | Tertile1, CMD ≥2 | 0.197(0.028,0.366) | 2.449e-02 | 0.220 | 0.179(-0.036,0.394) | 0.109 | 0.398 |
|  | Tertile2, CMD=0 | -0.003(-0.18,0.175) | 0.976 | 0.9897 | -0.031(-0.2,0.138) | 0.723 | 0.904 |
|  | Tertile2, CMD=1 | 0.171(0.02,0.322) | 2.785e-02 | 0.232 | 0.191(-0.014,0.395) | 7.092e-02 | 0.345 |
|  | Tertile2, CMD ≥2 | 0.057(-0.139,0.254) | 0.568 | 0.870 | 0.151(-0.058,0.359) | 0.162 | 0.476 |
|  | Tertile3, CMD=0 | 0.047(-0.135,0.229) | 0.613 | 0.874 | 0.034(-0.156,0.224) | 0.728 | 0.904 |
|  | Tertile3, CMD=1 | 0.143(-0.043,0.329) | 0.134 | 0.449 | 0.146(-0.035,0.327) | 0.116 | 0.398 |
|  | Tertile3, CMD ≥2 | 0.1(-0.114,0.314) | 0.363 | 0.712 | 0.238(-0.042,0.519) | 0.101 | 0.398 |
