# Supplementary material for: Peripheral and central auditory dysfunction, cardiometabolic multimorbidity, and cognitive performance in community-dwelling older adults: a cross-sectional study
Source: Front Neurosci. 2026 Jan 16;19:1646313. doi: 10.3389/fnins.2025.1646313 (PMC12856757; doi:10.3389/fnins.2025.1646313)
Supplement: Supplementary file 10 [file Table_9.docx]

Supplementary Table 8. The mediating effects of CMM on the relationship between LPTA, HPTA, or SNR and global cognitive performance in Model 2

| Groups |  | Total sample Model 2 | |  | Sensitivity test Model 2 | |  |
| --- | --- | --- | --- | --- | --- | --- | --- |
|  |  | β_Effect_ (95%CI) | P value | Adjusted P value | β_Effect_ (95%CI) | P value | Adjusted P value |
| Pre-MCI vs  Cognitively normal control | **High_Frq** |  |  |  |  |  |  |
|  | Undirect | 2.322e-04(-2.616e-04,8.560e-04) | 0.378 | 0.552 | 5.003e-04(-9.797e-06,1.385e-03) | 0.062 | 0.183 |
|  | Direct | 2.146e-03(-1.205e-03,3.939e-03) | 0.162 | 0.257 | 3.101e-03(-2.599e-04,4.666e-03) | 0.064 | 0.183 |
|  | Total | 2.379e-03(-8.836e-04,4.056e-03) | 0.126 | 0.228 | 3.602e-03(6.423e-04,4.906e-03) | 0.016 | 0.064 |
|  | Mediation Prop | 0.098(-0.515,1.045) | 0.436 | 0.592 | 0.139(-9.482e-03,0.894) | 0.074 | 0.185 |
| MCI vs Cognitively normal control | **High_Frq** |  |  |  |  |  |  |
|  | Undirect | 3.208e-04(-1.046e-04,8.860e-04) | 0.132 | 0.228 | 6.809e-04(9.675e-05,1.623e-03) | 0.01 | 0.05 |
|  | Direct | 3.808e-03(2.348e-03,4.550e-03) | 0 | 0 | 3.530e-03(1.508e-03,4.375e-03) | 0.006 | 0.05 |
|  | Total | 4.129e-03(2.781e-03,4.778e-03) | 0 | 0 | 4.211e-03(2.692e-03,4.897e-03) | 0 | 0 |
|  | Mediation Prop | 0.078(-0.028,0.236) | 0.132 | 0.228 | 0.162(0.026,0.493) | 0.01 | 0.05 |
| Pre-MCI vs MCI | **Low_Frq** |  |  |  |  |  |  |
|  | Undirect | -9.825e-05(-5.087e-04,2.127e-04) | 0.596 | 0.717 | 1.006e-04(-8.267e-04,1.102e-03) | 0.774 | 0.815 |
|  | Direct | 5.364e-03(1.453e-03,7.833e-03) | 0.008 | 0.0507 | 3.227e-03(-4.869e-03,8.457e-03) | 0.428 | 0.571 |
|  | Total | 5.265e-03(1.447e-03,7.746e-03) | 0.012 | 0.057 | 3.328e-03(-4.743e-03,8.465e-03) | 0.412 | 0.571 |
|  | Mediation Prop | -0.019(-0.134,0.049) | 0.604 | 0.717 | 0.030(-0.923,0.901) | 0.834 | 0.834 |
|  | **High_Frq** |  |  |  |  |  |  |
|  | Undirect | 1.756e-05(-2.143e-04,3.150e-04) | 0.856 | 0.856 | 2.029e-04(-2.225e-04,8.357e-04) | 0.406 | 0.571 |
|  | Direct | 3.158e-03(-2.034e-04,5.017e-03) | 0.068 | 0.171 | 2.088e-03(-1.988e-03,4.694e-03) | 0.316 | 0.527 |
|  | Total | 3.175e-03(-1.869e-04,5.024e-03) | 0.066 | 0.171 | 2.291e-03(-1.606e-03,4.776e-03) | 0.268 | 0.487 |
|  | Mediation Prop |  |  |  | 0.089(-0.796,1.273) | 0.59 | 0.656 |
|  | **SNR** |  |  |  |  |  |  |
|  | Undirect | -1.523e-04(-1.678e-03,8.426e-04) | 0.8 | 0.856 | -9.274e-04(-4.463e-03,1.269e-03) | 0.502 | 0.628 |
|  | Direct | 0.014(-1.019e-03,0.031) | 0.066 | 0.171 | 0.015(-6.133e-03,0.036) | 0.152 | 0.328 |
|  | Total | 0.014(-1.375e-03,0.030) | 0.072 | 0.171 | 0.014(-7.692e-03,0.035) | 0.164 | 0.328 |
|  | Mediation Prop | -0.011(-0.214,0.157) | 0.816 | 0.856 | -0.068(-0.687,0.287) | 0.578 | 0.656 |
