# Supplementary material for: Peripheral and central auditory dysfunction, cardiometabolic multimorbidity, and cognitive performance in community-dwelling older adults: a cross-sectional study
Source: Front Neurosci. 2026 Jan 16;19:1646313. doi: 10.3389/fnins.2025.1646313 (PMC12856757; doi:10.3389/fnins.2025.1646313)
Supplement: Supplementary file 11 [file Table_10.docx]

Supplementary Table 9. The mediating effects of CMM on the relationship between LPTA, HPTA, or SNR and domain-specific cognitive performance in Model 2

| Groups |  | Total sample Model 2 | |  | Sensitivity test Model 2 | |  |
| --- | --- | --- | --- | --- | --- | --- | --- |
|  |  | β_Effect_ (95%CI) | P value | Adjusted P value | β_Effect_ (95%CI) | P value | Adjusted P value |
| Processing by TMT A | **Low_Frq** |  |  |  |  |  |  |
|  | Undirect | 1.304e-05(-2.234e-04,2.569e-04) | 0.934 | 0.956 | 2.760e-04(-6.103e-05,9.512e-04) | 0.144 | 0.315 |
|  | Direct | 1.988e-03(2.379e-04,2.975e-03) | 0.028 | 0.129 | 2.479e-03(-6.806e-04,3.320e-03) | 0.098 | 0.242 |
|  | Total | 2.001e-03(1.975e-04,3.014e-03) | 0.034 | 0.147 | 2.755e-03(-2.960e-04,3.523e-03) | 0.054 | 0.177 |
|  | Mediation Prop | 6.518e-03(-0.215,0.201) | 0.924 | 0.956 | 0.100(-0.109,0.780) | 0.186 | 0.377 |
|  | **High_Frq** |  |  |  |  |  |  |
|  | Undirect | 8.330e-05(-3.445e-05,2.570e-04) | 0.196 | 0.594 | 1.683e-04(4.098e-06,5.051e-04) | 0.044 | 0.160 |
|  | Direct | 1.619e-03(6.729e-04,1.975e-03) | 0.008 | 0.046 | 1.496e-03(4.361e-04,1.872e-03) | 0.016 | 0.0736 |
|  | Total | 1.702e-03(8.622e-04,2.032e-03) | 0.002 | 0.0197 | 1.665e-03(7.407e-04,2.026e-03) | 0.008 | 0.050 |
|  | Mediation Prop | 0.049(-0.022,0.226) | 0.198 | 0.594 | 0.101(-4.236e-04,0.396) | 0.052 | 0.177 |
|  | **SNR** |  |  |  |  |  |  |
|  | Undirect | 1.681e-04(-1.199e-03,1.728e-03) | 0.754 | 0.956 | -3.374e-04(-2.376e-03,1.712e-03) | 0.77 | 0.902 |
|  | Direct | 6.145e-03(-3.664e-03,0.018) | 0.192 | 0.594 | 4.227e-03(-0.010,0.019) | 0.568 | 0.837 |
|  | Total | 6.313e-03(-3.711e-03,0.018) | 0.184 | 0.594 | 3.889e-03(-0.010,0.019) | 0.61 | 0.877 |
|  | Mediation Prop | 0.027(-0.444,0.570) | 0.766 | 0.956 | -0.087(-1.411,1.383) | 0.884 | 0.953 |
| Attention/executive function (by TMT B) | **Low_Frq** |  |  |  |  |  |  |
|  | Undirect | 2.347e-05(-3.802e-04,4.348E-04) | 0.888 | 0.956 | 6.604e-04(-1.202e-04,1.743e-03) | 0.092 | 0.235 |
|  | Direct | 1.070e-03(-1.624e-03,2.340e-03) | 0.352 | 0.714 | 1.429e-03(-3.446e-03,2.877e-03) | 0.482 | 0.773 |
|  | Total | 1.093e-03(-1.546e-03,2.390e-03) | 0.352 | 0.714 | 2.089e-03(-2.580e-03,3.356e-03) | 0.302 | 0.548 |
|  | Mediation Prop | 0.021(-1.513,1.287) | 0.9 | 0.956 | 0.316(-3.001,2.531) | 0.342 | 0.605 |
|  | **High_Frq** |  |  |  |  |  |  |
|  | Undirect | 1.432e-04(-5.857e-05,4.297e-04) | 0.17 | 0.594 | 2.876e-04(5.398e-05,6.886e-04) | 0.018 | 0.078 |
|  | Direct | 1.379e-03(3.357e-04,1.756e-03) | 0.022 | 0.108 | 1.378e-03(5.013e-04,1.764e-03) | 0.024 | 0.092 |
|  | Total | 1.522e-03(4.724e-04,1.873e-03) | 0.018 | 0.096 | 1.666e-03(8.765e-04,2.053e-03) | 0.006 | 0.050 |
|  | Mediation Prop | 0.094(-0.085,0.429) | 0.18 | 0.594 | 0.173(0.033,0.531) | 0.024 | 0.092 |
|  | **SNR** |  |  |  |  |  |  |
|  | Undirect | 2.496e-04(-1.585e-03,2.172e-03) | 0.778 | 0.956 | -5.780e-04(-3.400e-03,2.633e-03) | 0.746 | 0.902 |
|  | Direct | -1.006e-03(-9.407e-03,9.214e-03) | 0.856 | 0.956 | 1.177e-03(-0.011,0.016) | 0.916 | 0.972 |
|  | Total | -7.561e-04(-9.689e-03,9.660e-03) | 0.892 | 0.956 | 5.992e-04(-0.013,0.016) | 0.992 | 0.992 |
|  | Mediation Prop | -0.330(-2.262,2.066) | 0.854 | 0.956 | -0.965(-2.483,3.872) | 0.758 | 0.902 |
| Delayed recall (by HVLT-R) | **Low_Frq** |  |  |  |  |  |  |
|  | Undirect | 4.204e-06(-9.975e-05,1.072e-04) | 0.928 | 0.956 | 6.081e-05(-1.603e-04,3.831e-04) | 0.57 | 0.837 |
|  | Direct | 2.763e-03(1.932e-03,3.284e-03) | 0 | 0 | 3.234e-03(1.465e-03,3.838e-03) | 0.008 | 0.050 |
|  | Total | 2.767e-03(1.934e-03,3.292e-03) | 0 | 0 | 3.295e-03(1.619e-03,3.917e-03) | 0.008 | 0.050 |
|  | Mediation Prop | 1.519e-03(-0.039,0.039) | 0.928 | 0.956 | 0.018(-0.055,0.150) | 0.57 | 0.837 |
|  | **High_Frq** |  |  |  |  |  |  |
|  | Undirect | 3.434e-05(-5.509e-05,1.768e-04) | 0.456 | 0.850 | 7.550E-05(-1.548e-04,4.042e-04) | 0.482 | 0.773 |
|  | Direct | 1.705e-03(7.560e-04,2.047e-03) | 0.004 | 0.025 | 1.514e-03(-6.121e-04,2.027e-03) | 0.118 | 0.271 |
|  | Total | 1.740e-03(8.010e-04,2.085e-03) | 0.004 | 0.025 | 1.589e-03(-4.917e-04,2.092e-03) | 0.104 | 0.247 |
|  | Mediation Prop | 0.020(-0.040,0.121) | 0.456 | 0.850 | 0.048(-0.360,0.478) | 0.538 | 0.837 |
|  | **SNR** |  |  |  |  |  |  |
|  | Undirect | 8.335e-05(-7.571e-04,1.004e-03) | 0.878 | 0.956 | -1.749e-04(-1.810e-03,1.235e-03) | 0.824 | 0.902 |
|  | Direct | 0.018(7.611e-03,0.031) | 0 | 0 | 0.022(6.344e-03,0.041) | 0.004 | 0.046 |
|  | Total | 0.018(7.733e-03,0.031) | 0 | 0 | 0.021(5.655e-03,0.041) | 0.004 | 0.046 |
|  | Mediation Prop | 4.577e-03(-0.048,0.072) | 0.878 | 0.956 | -8.157e-03(-0.091,0.073) | 0.824 | 0.902 |
| Recognition (by HVLT-R) | **Low_Frq** |  |  |  |  |  |  |
|  | Undirect | 1.464e-05(-2.789e-04,2.917e-04) | 0.898 | 0.956 | 3.247e-04(-2.630e-04,1.094e-03) | 0.27 | 0.511 |
|  | Direct | -3.552e-04(-3.712e-03,2.788e-03) | 0.852 | 0.956 | 1.239e-03(-4.715e-03,6.416e-03) | 0.7 | 0.902 |
|  | Total | -3.405E-04(-3.745e-03,2.783e-03) | 0.868 | 0.956 | 1.564e-03(-4.388e-03,6.613e-03) | 0.626 | 0.882 |
|  | Mediation Prop | -0.043(-0.868,1.038) | 0.942 | 0.956 | 0.208(-2.391,1.535) | 0.708 | 0.902 |
|  | **High_Frq** |  |  |  |  |  |  |
|  | Undirect | 1.228e-04(-9.031e-05,4.246e-04) | 0.268 | 0.630 | 2.199e-04(-1.595e-04,7.154e-04) | 0.274 | 0.511 |
|  | Direct | 1.276e-03(-1.244e-03,3.220e-03) | 0.274 | 0.630 | 2.018e-03(-1.465e-03,4.275e-03) | 0.216 | 0.426 |
|  | Total | 1.399e-03(-1.064e-03,3.360e-03) | 0.24 | 0.630 | 2.238e-03(-1.246e-03,4.394e-03) | 0.182 | 0.377 |
|  | Mediation Prop | 0.088(-0.823,0.765) | 0.444 | 0.850 | 0.098(-0.756,0.690) | 0.412 | 0.711 |
|  | **SNR** |  |  |  |  |  |  |
|  | Undirect | 1.340e-04(-1.110e-03,1.415e-03) | 0.834 | 0.956 | -2.287e-04(-2.421e-03,1.143e-03) | 0.806 | 0.902 |
|  | Direct | 6.416e-03(-4.491e-03,0.020) | 0.24 | 0.630 | 0.016(-1.851e-03,0.036) | 0.074 | 0.213 |
|  | Total | 6.550e-03(-4.811e-03,0.020) | 0.246 | 0.630 | 0.016(-2.079e-03,0.036) | 0.078 | 0.215 |
|  | Mediation Prop | 0.020(-0.404,0.622) | 0.772 | 0.956 | -0.014(-0.280,0.160) | 0.808 | 0.902 |
| Language (by BNT) | **Low_Frq** |  |  |  |  |  |  |
|  | Undirect | -5.549e-08(-1.933e-05,1.457e-05) | 0.574 | 0.956 | 2.792e-06(-6.850e-07,4.212e-05) | 0.146 | 0.315 |
|  | Direct | 3.331e-04(0.000,1.676e-03) | 0.002 | 0.0197 | -3.658e-05(-1.704e-04,0.000) | 0.008 | 0.050 |
|  | Total | 3.330e-04(0.000,1.658e-03) | 0.002 | 0.0197 | -3.379e-05(-1.383e-04,0.000) | 0.016 | 0.072 |
|  | Mediation Prop | -1.666e-04(-0.049,0.049) | NA |  | -0.083(-0.624,0.051) | NA |  |
|  | **High_Frq** |  |  |  |  |  |  |
|  | Undirect | 9.645e-08(-8.725e-07,3.632e-06) | 0.478 | 0.868 | 6.224e-07(0.000,9.675e-06) | 0.01 | 0.058 |
|  | Direct | -1.092E-05(-5.270E-05,0.000) | 0.004 | 0.026 | -4.809e-06(-4.617e-05,0.000) | 0 | 0 |
|  | Total | -1.083e-05(-4.942e-05,0.000) | 0.004 | 0.025 | -4.187e-06(-3.612e-05,0.000) | 0 | 0 |
|  | Mediation Prop | -8.908e-03(-0.155,0.064) | NA |  | -0.149(-0.364,-5.389e-03) | NA |  |
|  | **SNR** |  |  |  |  |  |  |
|  | Undirect | 3.880e-07(-1.908e-05,4.672e-05) | 0.614 | 0.956 | -1.757e-05(-3.475e-04,1.555e-04) | 0.456 | 0.767 |
|  | Direct | 1.372e-04(-4.863e-04,7.147e-04) | 0.344 | 0.714 | -1.097e-03(-6.516e-03,0.000) | 0 | 0 |
|  | Total | 1.376e-04(-4.422e-04,7.066e-04) | 0.336 | 0.714 | -1.115e-03(-6.739e-03,0.000) | 0 | 0 |
|  | Mediation Prop | 2.820E-03(-0.473,0.352) | NA |  | 0.016(-0.099,0.110) | NA |  |
| Language (by animal fluency) | **Low_Frq** |  |  |  |  |  |  |
|  | Undirect | 1.733e-05(-3.172e-04,3.420e-04) | 0.894 | 0.956 | 7.295e-04(-7.847e-05,1.787e-03) | 0.088 | 0.234 |
|  | Direct | 1.469e-03(-1.202e-03,3.083e-03) | 0.256 | 0.630 | -4.778e-04(-7.805e-03,3.319e-03) | 0.818 | 0.902 |
|  | Total | 1.487e-03(-1.049e-03,3.087e-03) | 0.25 | 0.630 | 2.517e-04(-6.892e-03,3.858e-03) | 0.972 | 0.992 |
|  | Mediation Prop | 0.012(-0.794,0.668) | 0.904 | 0.956 | 2.898(-4.936,3.676) | 0.936 | 0.979 |
|  | **High_Frq** |  |  |  |  |  |  |
|  | Undirect | 1.644e-04(-7.099e-05,4.945e-04) | 0.182 | 0.594 | 5.502e-04(7.265e-05,1.225e-03) | 0.014 | 0.074 |
|  | Direct | 4.195e-04(-2.413e-03,1.875e-03) | 0.736 | 0.956 | -4.765e-05(-3.582e-03,1.938e-03) | 0.988 | 0.992 |
|  | Total | 5.839e-04(-2.176e-03,2.019e-03) | 0.618 | 0.956 | 5.025e-04(-2.670e-03,2.300e-03) | 0.746 | 0.902 |
|  | Mediation Prop | 0.281(-1.639,2.489) | 0.664 | 0.956 | 1.095(-7.760,5.073) | 0.752 | 0.902 |
|  | **SNR** |  |  |  |  |  |  |
|  | Undirect | 1.811e-04(-1.273e-03,1.671e-03) | 0.74 | 0.956 | -4.010e-04(-2.784e-03,2.119e-03) | 0.716 | 0.902 |
|  | Direct | -4.333e-04(-0.012,9.959e-03) | 0.936 | 0.956 | -0.015(-0.025,1.518e-03) | 0.072 | 0.213 |
|  | Total | -2.522e-04(-0.011,0.010) | 0.97 | 0.97 | -0.015(-0.025,1.091e-03) | 0.062 | 0.194 |
|  | Mediation Prop | -0.718(-1.892,1.462) | 0.914 | 0.956 | 0.027(-0.205,0.293) | 0.734 | 0.902 |
